# DNA stimulates the deacetylase SIRT6 to mono-ADP-ribosylate proteins with histidine repeats

**DOI:** 10.1016/j.jbc.2025.108532

**Published:** 2025-04-23

**Authors:** Nicholas J. Pederson, Katharine L. Diehl

**Affiliations:** Department of Medicinal Chemistry, University of Utah, Salt Lake City, Utah, USA

**Keywords:** posttranslational modification (PTM), sirtuins, DNA repair, DNA–protein interaction, ADP-ribosylation

## Abstract

Sirtuins are the NAD^+^-dependent class III lysine deacylases (KDACs). Members of this family have been linked to longevity and a wide array of different diseases, motivating the pursuit of sirtuin modulator compounds. Sirtuin 6 (SIRT6) is a primarily nuclear KDAC that deacetylates histones to facilitate gene repression. In addition to this canonical posttranslational modification “eraser” function, SIRT6 can use NAD^+^ instead to “write” mono-ADP-ribosylation (mARylation) on target proteins. This enzymatic function has been primarily associated with SIRT6’s role in the DNA damage response. This modification has been challenging to study because it is not clear under what precise cellular contexts it occurs, only a few substrates are known, and potential interference from other ADP-ribosyltransferases in cells, among other reasons. In this work, we used commercially available ADP-ribosylation detection reagents to investigate the mARylation activity of SIRT6 in a reconstituted system. We observed that SIRT6 is activated in its mARylation activity by binding to dsDNA ends. We further identified a surprising target motif within biochemical substrates of SIRT6, polyhistidine repeat tracts, which are present in several previously identified SIRT6 mARylation substrates. This work provides important context for SIRT6 mARylation activity, in contrast to its KDAC activity, and generates a list of new potential SIRT6 mARylation substrates based on the polyhistidine motif.

In mammals, there are seven sirtuin family members (SIRT1-7). Sirtuins are NAD^+^-dependent enzymes that possess deacylase and/or mono-ADP-ribosyltransferase activity (1, 2, 3). Sirtuins have been associated with longevity and implicated in numerous diseases, such as cancer and cardiovascular disease (2, 4, 5, 6, 7, 8). For example, SIRT6 overexpression leads to lifespan extension in mice and *drosophila* (9, 10, 11), while SIRT6 deficiency leads to detrimental cellular and organismal effects (6, 8). Interestingly, SIRT6 has been identified as a tumor suppressor in some cancers and as tumorigenic in others (6, 7, 10, 12, 13, 14, 15). Due to the roles of SIRT6 in numerous pathways and disease states, this enzyme has been studied extensively and this includes the pursuit of pharmacological inhibitors and activators (16, 17, 18).

SIRT6 is perhaps best known as a histone deacetylase that acts at H3K9ac, H3K18ac, H3K27ac, and other sites to compact chromatin and aid in the silencing of target genes (19, 20, 21, 22). SIRT6’s relatively low activity on acetylated peptides (21, 23, 24, 25) has led to an idea that SIRT6 alone possesses insufficient deacetylase activity (26, 27). However, biochemical and structural data show that SIRT6 binds to the nucleosome with high affinity, rendering it active toward this substrate (1, 19, 20, 22, 24, 28), providing a rationale for its low activity on histone peptides compared to the context of a nucleosome substrate. In addition, SIRT6 has been shown to bind non-nucleosomal DNA, or so-called “free DNA,” and its ability to bind DNA has been linked to its role in the DNA damage response (29, 30, 31). Moreover, a recent paper reported that binding to free DNA increases SIRT6 activity on acylated peptides (27).

In addition to its deacetylase function, SIRT6 can deacylate (32), defatty-acylate (25, 26), or mono-ADP-ribosylate (“mARylate”) (33, 34, 35, 36, 37, 38) various substrates. Much like PARP1/2-catalyzed poly-ADP-ribosylation, SIRT6-catalyzed mARylation is associated primarily with oxidative stress and DNA damage conditions (33, 34, 35, 36). Its reported substrates to date are itself (specific site not identified) (39), PARP1 (at K521) (33, 34), KAP1 (specific site not identified) (37), KDM2A (at R1019) (35), BAF170/SMARCC2 (at K312) (36), lamin A (specific site not identified) (38), and YY1 (at E216, E218, and/or K409) (40). In the case of PARP1, SIRT6 was reported to mARylate K521, which is within the automodification loop of PARP1, to facilitate recruitment of PARP1 to DNA breaks and to activate its poly-ADP-ribosylation activity (33, 34). SIRT6-catalyzed mARylation of the BAF chromatin remodeling complex subunit BAF170 was shown to recruit the remodeler complex to the enhancer region of an oxidative stress response gene to enable activation of this gene (36). Lamin A was first shown to activate SIRT6 deacetylation and mARylation activity in DNA repair (41) and then was later also shown to itself be mARylated by SIRT6 in cells to promote genome stability (38). It was recently shown that SIRT6 mARylates the transcription factor YY1, promoting its dissociation from DNA and subsequent degradation (40). Yet, relatively little is understood about what causes SIRT6 to mARylate substrates, what other substrates it might have, or what the function of this modification is. Here, we demonstrate in biochemical assays that free DNA promotes SIRT6 to mARylate its known target BAF170 (36) as well as two novel substrates MeCP2 and NLK. We demonstrate that SIRT6 mARylation activity is dependent on the presence of the histidine repeats within these proteins, although direct evidence of the precise site/s of modification remained elusive. We also show *in cellulo* that SIRT6’s activity on YY1 is dependent on the presence of YY1’s polyhistidine (“polyHis”) motif. Taken together, our data provide mechanistic insights into how SIRT6 is directed toward its mARylation activity and create a list of other potential SIRT6 mARylation substrates based on the “polyHis” motif.

## Results

### DNA promotes SIRT6 mARylation activity

**Figure 1:**
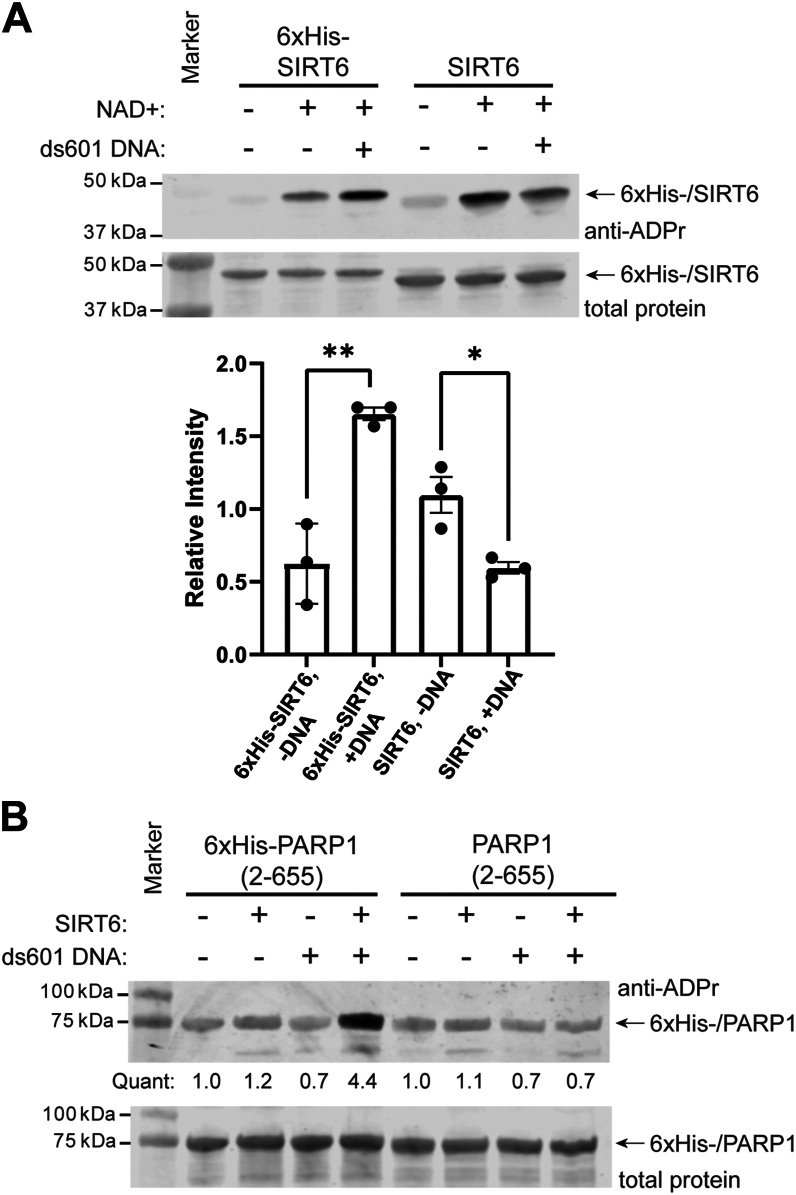
**DNA promotes SIRT6 mARylation activity.***A*, *Top:* immunoblot analysis of 6xHis-SIRT6 and SIRT6 (2 μM) auto-mARylation reactions with or without ds601 DNA (1 μM). The reactions were incubated at 37 °C for 2 h ± 1 mM NAD^+^. *Bottom:* quantification of the immunoblot data and replicate blots, n = 3. The densitometry was performed in Image Studio (Licor) with normalization to the SIRT6 band in the total protein stain. An unpaired, two-tailed *t* test was used: ∗∗*p* = 0.0034, ∗*p* = 0.0180, error bars show ± SD. *B*, immunoblot analysis of SIRT6 (10 μM) mARylation reactions with 6xHis-PARP1(2-655) or PARP1(2-655) (2 μM) as substrates. The reactions were incubated at 37 °C for 2 h with 1 mM NAD^+^ and ± 1 μM ds601 DNA, n = 2. mARylate, mono-ADP-ribosylate; mARylation, mono-ADP-ribosylation; SIRT6, sirtuin6.

Since SIRT6 binds to free DNA ends and is recruited to damaged DNA (29, 30, 31), we first tested if the addition of DNA would affect SIRT6 mARylation activity. We started by measuring SIRT6 autoactivity (39) with a biochemical ribosylation assay using a poly/mono-ADP-ribose antibody (Cell Signaling Technologies) for detection. We prepared two full-length (2–355), human SIRT6 proteins, one with an N-terminal 6xhistidine tag (“6xHis-SIRT6”) and another in which the 6xHis tag was proteolytically cleaved, rendering it identical to native human SIRT6 (“SIRT6,” residues S2–S355, Fig. S1). For the DNA, we used 177-bp dsDNA derived from the Widom 601 (42) sequence (“ds601,” sequence in the Supporting information). Strikingly, these enzymes respond differently to the presence of ds601 (Fig. 1*A*). The addition of ds601 significantly increased 6xHis-SIRT6 autoactivity compared to the absence of ds601. For SIRT6, the ds601 significantly decreased its autoactivity compared to the absence of ds601.This result was surprising since we initially reasoned the 6xHis tag might decrease enzyme activity given that the N terminus of SIRT6 is near the active site and is necessary for catalytic activity (21, 43).

Auto-mARylation by SIRT6 was shown previously to be an intramolecular (or “in *cis*”) reaction (39), so we next asked if other substrates would be affected since the intermolecular (or “in *trans*”) mARylation mechanism could differ. SIRT6 mARylation of PARP1 was shown to activate PARP1 and promote DNA damage repair in cells (33, 34). We generated the same hPARP1 fragment (PARP1(2-655)) that was used in the biochemical assays in the previous study (34). This truncation lacks the entire catalytic domain and cannot auto-PARylate and was reported to be mARylated by SIRT6 (at PARP1 K521). We made versions of this truncated PARP1 with and without the N-terminal 6xHis tag. We note that it was not described in the previous study if the PARP1(2-655) that was used contained a 6xHis tag or not (34). Surprisingly, we observed SIRT6 dependent modification of 6xHis-PARP1(2-655) only in the presence of dsDNA and no modification of PARP1(2-655) whether dsDNA was present or not (Fig. 1*B*).

### SIRT6 mARylates polyHis tract-containing proteins

Given the discrepancy we observed between SIRT6’s mARylation behavior toward the 6xHis *versus* the tag-less proteins, we wondered if the exogenous histidine tag on SIRT6 and PARP1(2-655) could mimic a motif in endogenous SIRT6 substrates. PolyHis tracts are relatively uncommon in the human proteome compared to other single amino acid repeat tracts (44, 45). Most polyHis-containing proteins are annotated as nuclear proteins and many are transcription factors (44, 46, 47, 48). Interestingly, when we looked at the polyHis proteins in humans (Data File S1), we identified four proteins that are known to interact with SIRT6: BAF170 (also called SMARCC2) (36), lamin A/C (38, 41), MeCP2 (24), and YY1 (49) (Table 1). BAF170 (36), lamin A/C (38), and YY1 (40) are reported substrates of mARylation by SIRT6. MeCP2 (24) is reported to form a complex with SIRT6 in a DNA-dependent manner but is not known to be a mARylation substrate of SIRT6. Given this information, we next tested if SIRT6 would mARylate three different polyHis-containing proteins.

**Table 1:** SIRT6-associated and/or polyHis proteins with relevance to this study

Protein	UniProt	Association with SIRT6	Previously reported to be mAR target of SIRT6?	mARylated in this study by SIRT6?	PolyHis sequence	Number of total residues in the protein
PARP1	P09874	DNA damage (33, 34)	Yes	No^a^	None	1014
MeCP2	P51608	DNA damage (24)	No	Yes	366-HHHHHHH-372	486
BAF170	Q8TAQ2	DNA damage (36)	Yes	Yes	1117-HGHHHH-1122	1214
NLK	Q9UBE8	None	No	Yes	18 His in first 54 residues	527
LAMA	W8QEH3	Longevity (38, 41)	Yes	Not Tested	563-HHHH-566	572
GATA6	Q92908	DNA damage (76)	No	Not Tested	324-HHHHHHHHHH-333	595
YY1	P25490	Longevity (49), Transcription (40)	Yes	Yes	70-HHHHHHHHHHH-80	414

a PARP1(2-655) lacking a 6xHis tag was not mARylated by SIRT6 in our assay.

**Figure 2:**
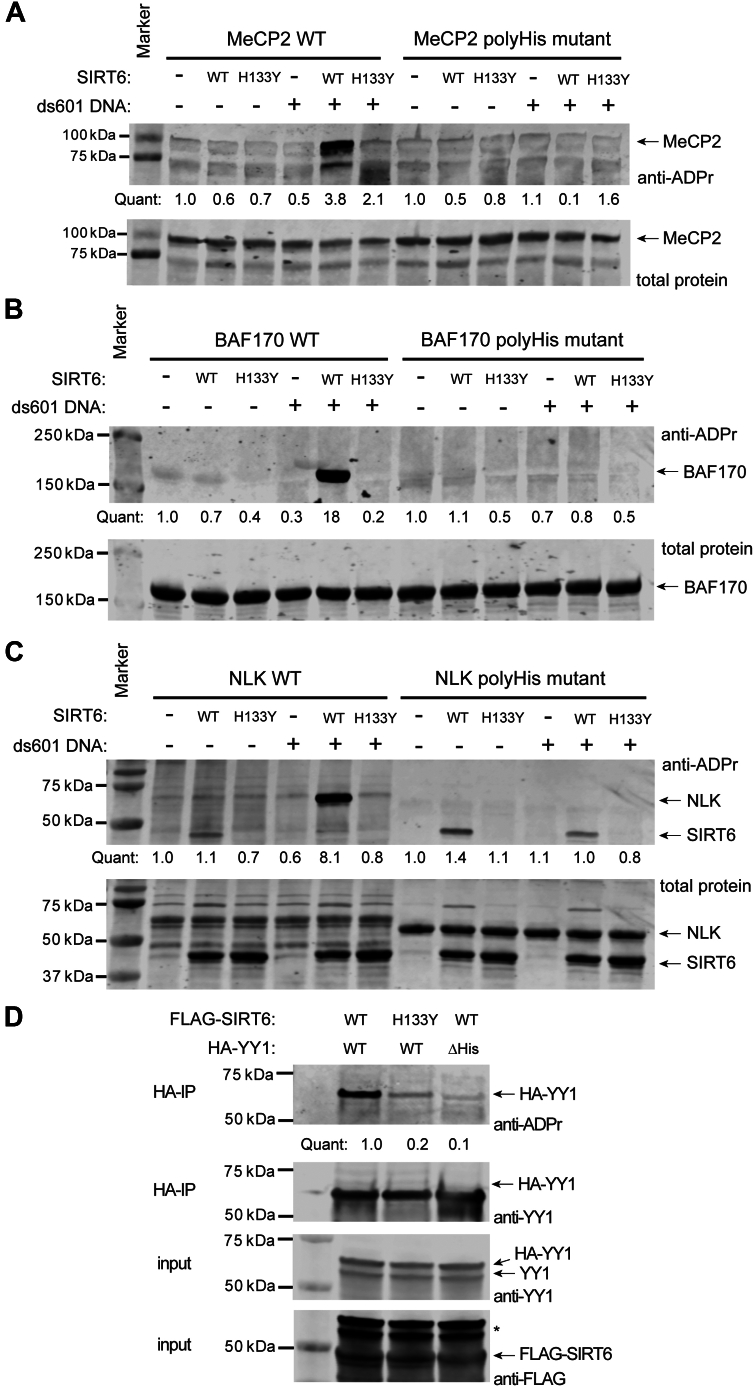
**SIRT6 mARylates polyHis tract-containing proteins.***A*–*C*, immunoblot analyses of SIRT6 (2 μM) mARylation reactions with (*A*) MeCP2-WT or MeCP2 polyHis mutant, (*B*) BAF170-WT or BAF170 polyHis mutant, or (*C*) NLK-WT or NLK polyHis mutant as the substrate (all at 2 μM). SIRT6-H133Y is an inactive mutant that was included as a negative control. The reactions in *panels A* and *C* were incubated at 37 °C for 2 h and in *panel B* for 20 min with 1 mM NAD^+^ and ± 1 μM ds601 DNA, n = 2. The immunoblots in (*A–C*) were performed using a poly/mono-ADP-ribose antibody (Cell Signaling Technologies #83732). *D*, immunoblot analysis of immunoprecipitated HA-YY1-WT or HA-YY1-ΔpolyHis from HEK293T cells expressing the respective YY1 protein and either FLAG-SIRT6-WT or FLAG-SIRT6-H133Y. The anti-ADPr blot was obtained used a mono-ADP-ribose–specific binding reagent (Millipore #MABE-1076), n = 2. mARylate, mono-ADP-ribosylate; mARylation, mono-ADP-ribosylation; polyHis, polyhistidine; SIRT6, sirtuin6.

We chose MeCP2, BAF170, and nemo-like kinase (NLK) as substrates for mARylation assays with SIRT6. NLK was chosen as a polyHis protein with no prior association with SIRT6 or ADP-ribosylation. To avoid using a 6xHis tag for purification, we used a glutathione-*S*-transferase tag to purify MeCP2 from *Escherichia coli*, and we used a FLAG tag to purify BAF170 and NLK from *Spodoptera frugiperda* (Sf9). We purified the full-length MeCP2 (2-486), BAF170 (1-1214), and NLK (1-527) as well as mutants of each lacking the histidine repeats (the full amino acid sequences are provided in the Supporting information). For MeCP2 and BAF170, the polyHis tracts were mutated to glycines. For NLK, which has two polyHis tracts in the N terminus, we truncated the N terminus (Δ2-54) and mutated H124/H125/H126 to alanine. SIRT6 mARylates MeCP2, BAF170, and NLK only in the presence of dsDNA and is unable to mARylate the polyHis mutants (Fig. 2, *A*–*C*). We used a catalytically inactive mutant of SIRT6 (SIRT6-H133Y (43)) to confirm that the mARylation was dependent on the enzymatic activity of SIRT6 (Fig. 2, *A*–*C*). We note that BAF170 mARylation was obvious within a 20 min reaction (Fig. 2*B*), while MeCP2 and NLK mARylation required a longer incubation time to be apparent (2 h, Fig. 2, *A* and *C*). We tested the same reaction conditions with MeCP2 using a pan-ADP-ribose binding reagent (Millipore) that is derived from a bacterial macrodomain (50) for detection (Fig. S2*A*). The same results were obtained with both the antibody and macrodomain detection methods (51).

A recent paper reported that SIRT6 mARylates the transcription factor YY1 in human cells (40). We similarly coexpressed FLAG-SIRT6-WT or FLAG-SIRT6-H133Y with HA-YY1-WT in HEK293T cells and immunoprecipitated the YY1 to assess mARylation of it using a mono-ADP-ribose–specific binding reagent (Millipore). We recapitulated the SIRT6-dependent mARylation of YY1 (Fig. 2*D*). We coexpressed FLAG-SIRT6-WT and HA-YY1-ΔpolyHis in the cells (HEK293T or HepG2) and observed no mARylation of the HA-YY1-ΔpolyHis (Figs. 2*D* and S2*B*), which is consistent with our other biochemical data for MeCP2, BAF170, and NLK. Altogether, these results support our initial finding that DNA stimulates SIRT6 to mARylate and that this happens on substrates containing histidine repeats. This also identifies MeCP2, NLK, and other polyHis proteins as potential new SIRT6 mARylation substrates and demonstrates that the polyHis motif in YY1 is important for its mARylation in cells.

### SIRT6 mARylation activity is activated by binding to DNA ends

**Figure 3:**
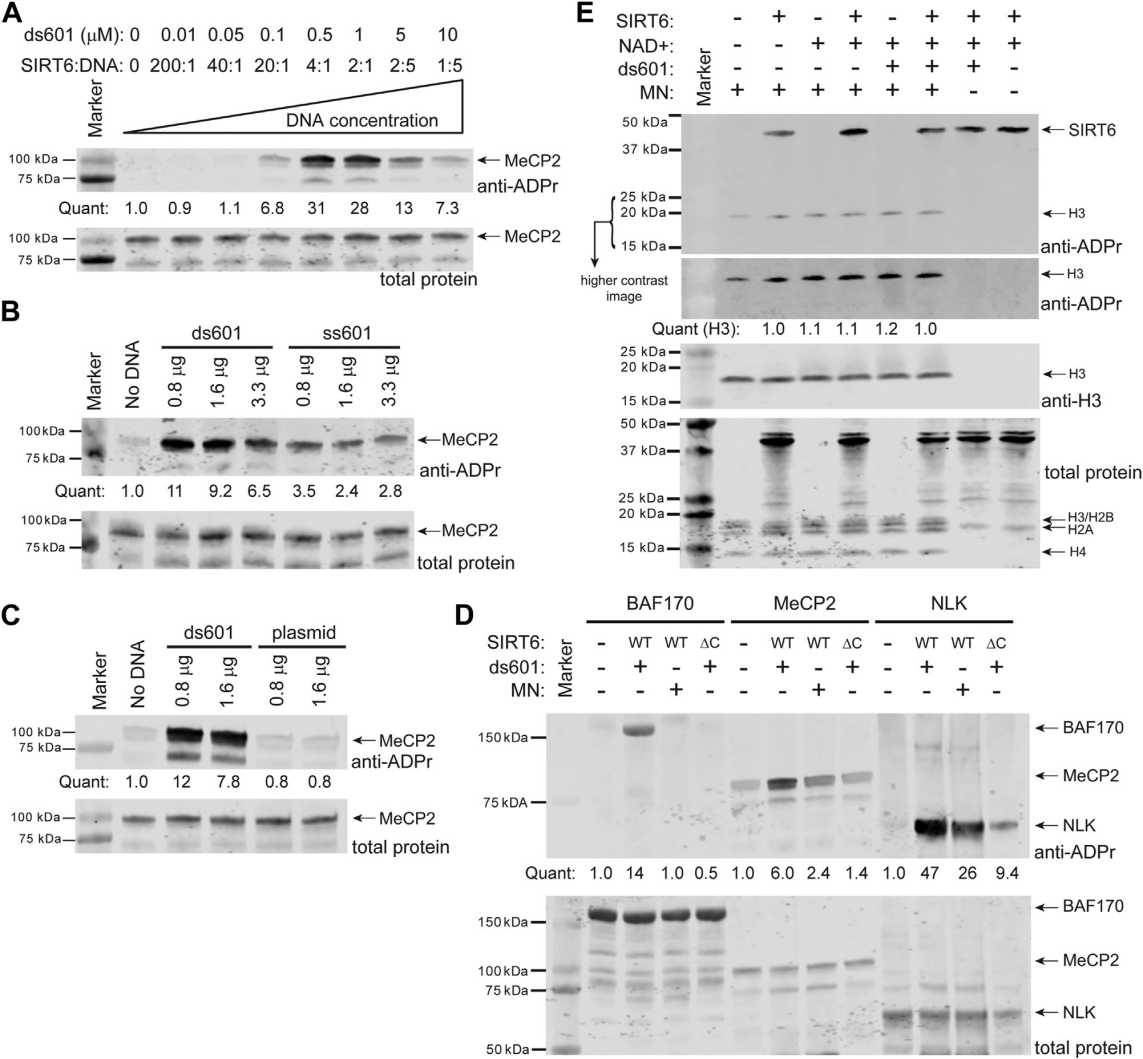
**SIRT6 mARylation activity is activated by binding to DNA ends.***A*, titration of ds601 in the MeCP2 (2 μM) mARylation assay by SIRT6 (2 μM), n = 2. *B*, titration of ds601 and ss601 in the MeCP2 (2 μM) mARylation assay by SIRT6 (2 μM), n = 2. Note that there was no SIRT6 in the reaction for the “no DNA” lane. In terms of concentration, 0.8 μg = 0.5 μM ds601, 1.6 μg = 1 μM ds601, and 3.3 μg = 2 μM ds601. *C*, immunoblot analysis of mARylation by SIRT6 (2 μM) of MeCP2 (2 μM) in the presence of plasmid DNA (pET28a), n = 2. Note that there was SIRT6 in the reaction for the “no DNA” lane. *D*, immunoblot analysis of mARylation assays of SIRT6-WT or -ΔC (2 μM) with ds601 or WT mononucleosome (1 μM) with BAF170, MeCP2, or NLK (2 μM) as the substrate, n = 2. The BAF170 reactions were incubated for 20 min. *E*, immunoblot analysis of WT nucleosomes (200 nM) incubated with SIRT6-WT (2 μM), ds601 (1 μM), and 1 mM NAD + at 37 °C for 2 h, n = 2. The poly/mono-ADP-ribose antibody (Cell Signaling Technologies #83732) was used for all the blots shown here. All the mARylation assays were incubated at 37 °C for 2 h unless otherwise noted. mARylate, mono-ADP-ribosylate; mARylation, mono-ADP-ribosylation; polyHis, polyhistidine; SIRT6, sirtuin6; NLK, nemo-like kinase.

SIRT6 plays a role in the DNA damage response and was shown to promote nonhomologous end joining and homologous recombination in cells (29, 52). SIRT6 was also shown to independently arrive at DNA double strand breaks in cells (29, 30, 31). Previous work showed that SIRT6 binds to the ends of free DNA and that it binds as a dimer to the DNA end (*i.e.*, one molecule of SIRT6 on each strand end) (29, 30). We performed a DNA titration to determine how the binding stoichiometry would affect SIRT6 activity (Fig. 3*A*). We used the ds601 DNA as before and MeCP2 as the substrate. The data show three regimes: (1) at low concentrations of DNA (0.01–0.1 μM ds601), the affinity between the SIRT6 and the DNA (*i.e.*, K_d_ of around 1 μM) (29, 30) is too weak for binding to occur and mARylation of MeCP2 is low, (2) at 4:1 and 2:1 SIRT6:DNA (= 0.5 and 1 μM ds601) mARylation of MeCP2 is maximal, and (3) at 2:5 and 1:5 SIRT6:DNA (*i.e.*, DNA in excess) mARylation of MeCP2 is actually lower. The presence of this third regime indicates that the occupancy of SIRT6 (and/or MeCP2) on a given molecule of DNA is important for the reaction. One possible explanation for this behavior is that the “mARylation active” form of SIRT6 is the DNA end–bound dimer. Under excess DNA, two molecules of SIRT6 are unlikely to bind at the same DNA end, so the mARylation activity observed is low. Another possible explanation is that the DNA brings the SIRT6 and MeCP2 in proximity to facilitate the mARylation reaction. Similarly, an excess of DNA would lead to a low likelihood that a molecule of SIRT6 and of MeCP2 are bound to the same molecule of DNA. MeCP2 is itself a DNA-binding protein (53) with a K_d_ = 2 nM for unmethylated CpG DNA (for full-length MeCP2) (54). The ds601 DNA we used has more than 10 unmethylated CpG sites in it, so the reported K_d_ is a reasonable estimate for the interaction between our MeCP2 and ds601 DNA. It is important to note that the polyHis tract in MeCP2 lies in its C-terminal domain-β, which is not itself a DNA-binding domain but may influence the behavior of MeCP2’s DNA-binding domains (55). Indeed, deletion of C-terminal domain-β led to a 5-fold decrease in the K_d_ of MeCP2 (K_d_ ∼ 10 nM for unmethylated CpG DNA) (54). However, since the assays in Figure 2*A* were performed at micromolar concentrations of SIRT6, MeCP2, and ds601, we would not expect a difference in K_d_ of 2 nM *versus* 10 nM to be significant for MeCP2-WT *versus* MeCP2-polyHis mutant binding to ds601. Given all of this information, we do not believe the role of the polyHis tract in MeCP2 (or by extension the other polyHis substrates) is to recruit it to the ds601 DNA.

We next tested other dsDNAs (the 147 bp Widom ds601 sequence and sheared salmon sperm DNA) and also observed activation of mARylation (Fig. S3, *A* and *B*). We tested single-stranded 601 DNA (ssDNA) and observed a lower magnitude of activation than ds601 (Fig. 3*B*). We also tested circular plasmid DNA and found that it did not activate mARylation by SIRT6 (Fig. 3*C*). These findings are consistent with previous studies of SIRT6 DNA showing that it binds to DNA ends (27, 29, 30).

Various point mutations in the catalytic domain have been reported to decrease SIRT6’s DNA binding affinity (29). The C-terminal extension (residues 269–355) of SIRT6 is reported to contribute significantly to DNA binding (28). C-terminal truncations of SIRT6 have reduced deacetylase activity on nucleosomes (Fig. S3*C*) but deacetylate peptides in our (Fig. S3*D*) and others’ hands (28, 43). We compared the binding of SIRT6-WT and a SIRT6 C-terminal truncation (SIRT6-Δ298–355, abbreviated as “SIRT6-ΔC”) to the ds601 DNA by gel shift assay. Our results recapitulated prior findings (27, 28) in that both proteins bind the ds601 DNA, but the binding of the SIRT6-ΔC is weaker than the WT. The SIRT6-WT has a higher affinity for the DNA and exhibits a larger shift beginning at the 2.0 μM SIRT6 concentration that is not observed with the SIRT6-ΔC up to 7 μM protein (Fig. S3*E*). The multiple banding pattern in the SIRT6-WT assay is consistent with a higher SIRT6:DNA stoichiometry than 1:1. We then compared the mARylation activity of SIRT6-WT to -ΔC (both without a 6xHis tag) and found that the truncation retains some autoactivity (Fig. S3*F*). The truncation is impaired in its mARylation of BAF170, MeCP2, and NLK compared to SIRT6 WT (Fig. 3*D*).

SIRT6 deacetylates histone H3 lysine 9 acetylated (“H3K9ac”) mononucleosomes (Fig. S3*C*) (24). Cryo-EM structures of the nucleosome-bound SIRT6 show that it binds in a specific mode to the nucleosome surface, positioning it to place the H3 tail in the active site (19, 20, 22). If SIRT6’s mARylation activity were a completely nonspecific reaction driven simply by proximity to a nucleophilic residue (56), we would expect nucleosomal histones, particularly H3, to be readily modified as they contain many lysines, arginines, and other nucleophilic residues. While it was shown before that SIRT6 does not mARylate non-nucleosomal histones (39), we tested if SIRT6 would mARylate histones in the context of nucleosomes (Fig. 3*E*). We did not observe that SIRT6 mARylates histones (H3, H4, H2A, H2B) within nucleosomes. This result supports that proximity alone is insufficient for SIRT6 to mARylate proteins nonspecifically. Next, we wanted to know if nucleosomes could activate SIRT6 to mARylate our previously characterized substrates. We note that the nucleosomes we used have 15 bp “overhangs” on both sides of the Widom 601 sequence, which extend from the nucleosome core particle and are sufficient to activate PARP1 (57). Thus, SIRT6 could bind to those DNA ends as well, although its affinity for the nucleosome is higher (low micromolar for DNA ends (29) *versus* low nanomolar for nucleosomes (19, 28)). For BAF170, there was no detectable activation of SIRT6. For MeCP2 and NLK, there was some activation detected, although it was attenuated compared to ds601 (Fig. 3*D*). This result makes sense based on SIRT6’s high affinity for the nucleosome. In other words, if SIRT6 is bound to a nucleosome, it cannot simultaneously bind to and mARylate a polyHis substrate.

### The polyHis tract is required for SIRT6 to mARylate the proteins tested

**Figure 4:**
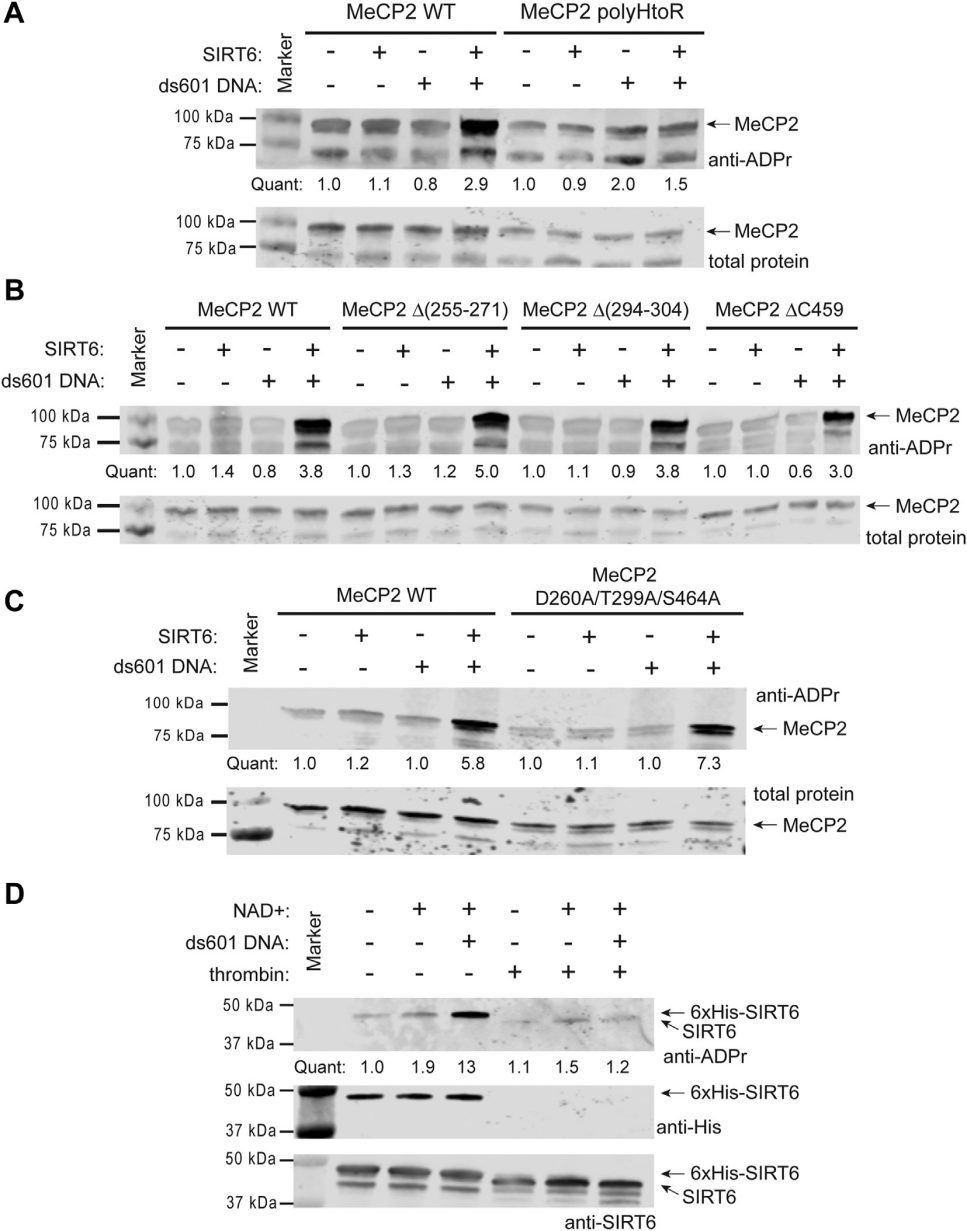
**The polyHis tract is required for SIRT6 to mARylate the proteins tested.***A*, immunoblot analysis of SIRT6 (2 μM) mARylation reactions with MeCP2-WT or -polyHtoR mutant (2 μM) with or without 1 μM ds601 (37 °C for 2 h), n = 2. *B*, immunoblot analysis of SIRT6 (2 μM) mARylation reactions with MeCP2-WT, (Δ255–271), (Δ294–304), or ΔC459 (2 μM) with or without 1 μM ds601 (37 °C for 2 h), n = 2. *C*, immunoblot analysis of SIRT6 (2 μM) mARylation reactions with MeCP2-D260A/T299A/S464A (2 μM) with or without 1 μM ds601 (37 °C for 2 h), n = 2. *D*, immunoblot analysis of 6xHis-SIRT6 (2 μM) mARylation (1 μM ds601, 1 mM NAD+, 37 °C for 20 min) before and after cleavage by thrombin (1 unit), n = 2. The poly/mono-ADP-ribose antibody (Cell Signaling Technologies #83732) was used for all the blots shown here. mARylate, mono-ADP-ribosylate; mARylation, mono-ADP-ribosylation; polyHis, polyhistidine; SIRT6, sirtuin6.

While it was clear from the mutagenesis data that a polyHis tract is required in PARP1(2-655), MeCP2, BAF170, NLK, and YY1 (Figs. 1 and 2) for SIRT6 to mARylate them, we next sought more details about the role of the polyHis tract. We created an MeCP2 construct with the histidine repeats mutated to arginine (“polyArg”). If the polyHis functions as a generic polycation for protein–protein or protein-DNA binding, then polyArg would serve the same role as polyHis (58, 59). Since arginine is mARylated by some ADP-ribosyltransferases (60), including SIRT6 (35), we reasoned that SIRT6 might be able to modify this MeCP2 construct. However, SIRT6 does not mARylate the polyArg MeCP2 (Fig. 4*A*). Moreover, SIRT6 will not mARylate the lysine- and arginine-rich histone H3 tail of a nucleosome (Fig. 3*E*) to which it is known to bind with high specificity (19, 20, 28). We next wanted to determine how a polyHis peptide would affect the mARylation of MeCP2. We titrated a polyHis peptide (HHHHHHGGG) into the MeCP2 mARylation assay and observed that the peptide inhibits mARylation by SIRT6 (Fig. S4*A*). This finding tends to rule out a mechanism of allosteric activation of SIRT6 by polyHis since the peptide acted as an inhibitor when added in *trans* rather than as an activator.

Mono-ADP-ribosylation has been identified on different nucleophilic amino acids including glutamate, aspartate, serine, lysine, arginine, and cysteine and there are known enzymes responsible for these modifications (61). We next sought to ascertain the site/s of modification by SIRT6 in the polyHis substrate context and considered the possibility that histidine itself could be modified. The mARylation of histidine has only recently been reported as occurring in human cells (62, 63). We analyzed the mARylated MeCP2 by mass spectrometry to directly observe the modified residues. The analysis indicated mARylation at three MeCP2 residues: D260, T299, and S464. We generated deletions (Δ255-271, Δ294-304, and a C-terminal deletion Δ459) that correlated to the entire tryptic peptides that were identified as being mARylated, in case the specific residue assignment was not accurate within the peptide, a common issue with trying to assign ADP-ribosylation sites (62, 64). These deletion proteins were all robustly mARylated by SIRT6 (Fig. 4*B*), unlike the polyHis mutant (Fig. 2*A*). We next produced a triple point mutant (MeCP2 D260A/T299A/S464A), and it was also mARylated by SIRT6 to the same extent as the WT substrate (Fig. 4*C*). This finding indicates that D260, T299, and S464 cannot be the only sites in MeCP2 that are mARylated by SIRT6. Unfortunately, the polyHis tract was in the 15% of the MeCP2 sequence that was not covered in the analysis (Fig. S4*B* and Data File S2), so it is yet possible that the polyHis tract itself and/or adjacent sites are modified. Thus, the mass spectrometric and mutagenesis data together were inconclusive as to the predominant site/s of mARylation in MeCP2.

We looked at the sequences surrounding the polyHis tracts in the three substrates that we tested and in the full set of 129 human polyHis proteins (Data File S1). There are no consensus motifs present in terms of other amino acids in the flanking sequences that could act as acceptor residues for the mARylation (Fig. S4, *C* and *D*). Even so, serine was one of the most common amino acids near the polyHis tracts, and it appears near the polyHis tract in several of the substrates we tested (Fig. S4*C*). To determine if SIRT6 could be primarily modifying serine/s in MeCP2, we used the serine ADP-ribosyl-hydrolase ARH3 (65, 66, 67). Incubation of ARH3 after the ADP-ribosylation reaction of MeCP2 resulted in no decrease in the degree of modification of the MeCP2 (Fig. S4*E*), indicating that the ADP-ribosylation is not primarily on serine/s (or tyrosines (68)) We further used hydroxylamine treatment, which removes only Glu-/Asp-linked ADP-ribosylation (69, 70), on the MeCP2 reactions. We observed no decrease in the modification of MeCP2, indicating that the ADP-ribosylation is not solely on glutamate and/or aspartate (Fig. S4*F*). Thus, it seems unlikely that the predominant SIRT6-catalyzed mARylation on MeCP2 is linked *via* a serine, tyrosine, glutamate, or aspartate. Nevertheless, there are still plenty of other potential acceptor residues: lysine, arginine, histidine, threonine, cysteine, asparagine, and glutamine (and many combinations thereof).

We next utilized our initial finding that auto-mARylation of the 6xHis-tagged SIRT6 is stimulated by DNA (Fig. 1*A*). This SIRT6 construct has a thrombin cleavage site (“LVPR|GS”) between the 6xHis tag and the beginning of the SIRT6 sequence. We performed the mARylation reaction followed by cleavage with thrombin. The ADP-ribosylation signal on the SIRT6 subsequently treated with thrombin was abolished compared to the SIRT6 not treated with thrombin (Fig. 4*D*). This result indicates that the predominant site/s of mARylation reside within or nearby in primary sequence to the polyHis tract in 6xHis-SIRT6 within the cleaved portion of the protein: GSSHHHHHHSSGLVPR. This sequence contains serine, histidine, and arginine as possible polyHis-dependent sites of modification for the 6xHis-SIRT6. Further mass spectrometric studies will be needed to fully resolve the site/s of SIRT6-catalyzed mARylation in MeCP2 and other polyHis proteins.

## Discussion

Sirtuins exhibit multiple enzymatic functions, and it has remained unclear to what extent the mARyltransferase activity that some sirtuins display is a “side reaction” to their canonical deacylase activity (56). Here we show that SIRT6 mARylation activity is robustly activated by the presence of linear DNA toward substrates containing a polyHis tract. The importance of DNA binding for this activity of SIRT6 is consistent with previous work showing that SIRT6 binds specifically to DNA ends and is rapidly recruited to DNA damage sites (29, 30, 31) and that its mARyltransferase activity is associated with oxidative stress and DNA damage conditions (33, 34, 35, 36). It is still unclear what drives SIRT6 to be recruited to DNA breaks in cells. It was shown that SIRT6 arrives at DNA breaks independently of MRE11, Ku80, or PARP1/2 (29), suggesting that SIRT6 directly recognizes breaks similarly to PARP1/2. However, SIRT6 binds to the nucleosome with low nanomolar affinity (19, 28) and to “free” DNA ends with high nanomolar (30) to low micromolar affinity (29). In contrast, PARP1 (zinc fingers F1 and F2) binds to a dsDNA break with low nanomolar affinity and to circular or unbroken DNA with low micromolar affinity (71). Given this information, it is unclear how SIRT6 could spontaneously reorganize from nucleosomes to DNA ends on its own. Further investigation is needed to understand this mechanism.

In this study, we observed that SIRT6 does not mARylate histones within nucleosomes even though it binds to them with high specificity/affinity and engages the H3 tail in its active site. This finding suggests that the mARyltransferase activity of SIRT6 is not driven simply by proximity to a nucleophilic residue but rather that there is a specific context in which this reaction occurs. Indeed, in all of the proteins we tested, SIRT6 would only mARylate the protein if it had a polyHis tract and if linear DNA was also present in the reaction. Nevertheless, the precise role of the polyHis tract remains unclear. We demonstrated that it is not likely to be an allosteric activator of SIRT6 (Fig. S4*A*). SIRT6 (27, 28, 29), PARP1 (71), MeCP2 (55), and YY1 (72) contain well-characterized DNA-binding domains, and the polyHis tract does not lie within these domains. Indeed, previous domain dissection of MeCP2 (54, 55) and YY1 (72) showed that the polyHis tract is dispensable for DNA binding. PARP1(2-655) contains zing fingers F1 and F2 that bind to DNA ends with high affinity (71). Given this information and our data, it seems less likely that the polyHis tract’s role is to drive substrate binding to the dsDNA to bring it in generic proximity to the DNA-bound SIRT6. It seems more likely that the polyHis interacts directly with SIRT6, but further studies are needed to learn more about the interactions.

While we tested a small subset of polyHis proteins in this study, this polyHis motif now provides a candidate list of many other potential SIRT6 mARylation substrates. This work also provides important clues about the context in which SIRT6 mARylation occurs that will be useful in studying this activity in cells. Indeed, we showed that YY1’s polyHis motif is important for it SIRT6-dependent mARylation in human cells. The regulatory purpose of SIRT6-catalyzed mARylation of these polyHis proteins is an area for further investigation. The histidine repeat tracts occur almost exclusively in disordered regions and often occur along with other repetitive tracts (*e.g.*, glutamine, proline) (44). It was shown that mutation or removal of polyHis tracts, such as in YY1 (46), inhibits localization of the protein to nuclear speckles and inhibits phase separation (44). For YY1, its polyHis tract is important for its phase separation behavior, subnuclear localization, recruitment of coactivators, and enhancer clustering in cells (46). Thus, this modification could be related to the liquid–liquid phase separation behavior of these polyHis proteins. Delineating the full scope of SIRT6’s biochemistry will be critical in clarifying its many cellular roles. This understanding is of particular importance in disease states in which SIRT6 or its substrates behave aberrantly and in considering the therapeutic potential of small molecule inhibitors or activators of SIRT6 which could possibly be used to modulate the enzymatic duality of SIRT6 (16, 17, 18).

## Experimental procedures

### Materials

All salt, buffers, and other chemicals were obtained from Thermo Fisher Scientific unless otherwise noted. Chemicals used for solid-phase peptide synthesis were obtained from Chem-Impex International unless otherwise noted. Other reagents for cloning, protein purification, and the biochemical assays are listed in Table 2. SIRT6 was a gift from Cheryl Arrowsmith (Addgene plasmid #41565; http://n2t.net/addgene:41565;RRID:Addgene_41565). pFastBac1 Flag Baf170 was a gift from Robert Kingston (Addgene plasmid #1955; http://n2t.net/addgene:1955; RRID:Addgene_1955) (73). PcDNA3.1 HA-YY1 was a gift from Richard Young (Addgene plasmid # 104395; http://n2t.net/addgene:104395; RRID:Addgene_104395) (74). The pACeBac1-PARP1 plasmid was generated in a previous study (57). The MeCP2 and NLK genes were synthesized as GeneBlocks by Integrated DNA Technologies.

**Table 2:** Materials

Reagent	Manufacturer	Catalog number
Q5 High-Fidelity DNA Polymerase	New England Biolabs (NEB)	M0491
Dpn1	New England Biolabs (NEB)	R0176
NEBuilder HiFi DNA Assembly Master Mix	New England Biolabs (NEB)	E2621
T4 PNK	New England Biolabs (NEB)	M0201
T4 DNA ligase	New England Biolabs (NEB)	M0202
6× NEB Purple DNA loading dye	New England Biolabs (NEB)	B7025
100 bp ladder	New England Biolabs (NEB)	N0551
1 kb ladder	New England Biolabs (NEB)	N3232
DH5α Mach1 competent cells	Invitrogen	C862003
MAX Efficiency™ DH10Bac Competent Cells	Gibco	10361012
Rosetta(DE3) Competent cells- Novagen	Sigma	70954
QG buffer	Qiagen	19063
Miniprep buffers and columns	Qiagen	27104
Protease Inhibitor Mini Tablets	Pierce	A32953
High Affinity Ni-Charged Resin	GenScript	L00223
Glutathione Resin	GenScript	L00206
Anti-DYKDDDDK (FLAG) G1 Affinity Resin	GenScript	L00432
DYKDDDDK Peptide	GenScript	RP10586
HiLoad Superdex 16/600 200pg column	Cytiva	28989335
Superdex 200 Increase 10/300 GL	Cytiva	28990944
Thrombin	Cytiva	27084601
Dialysis Tubing	Thermofisher	88244
XBridge BEH C18 columns	Waters	186003624, 186008193, 186003673
NAD^+^	New England Biolabs (NEB)	B9007S
Rabbit anti-poly/mono-ADP-ribose	Cell Signaling Technologies (CST)	83732^a^ (Lot 5)
Rabbit anti-H3K9ac	Cell Signaling Technologies (CST)	9649 (Lot 13)
Mouse anti-His Tag	Cell Signaling Technologies (CST)	2366 (Lot 14)
Rabbit anti-pan-ADP-ribose binding reagent	Millipore	MABE1016 (Lot 3595120)
Rabbit anti-mono-ADP-ribose binding reagent	Millipore	MABE-1076
Mouse anti-H3	Cell Signaling Technologies (CST)	3638 (Lot 11)
Rabbit anti-SIRT6	Cell Signaling Technologies (CST)	12486 (Lot 3)
Mouse anti-FLAG	Cell Signaling Technologies (CST)	9A3
Anti-HA magnetic beads	Thermo Fisher	88836
Mouse anti-YY1	Santa Cruz	7341
IRDye 680CW goat anti-mouse	LI-COR	926-68070
IRDye 800CW goat anti-rabbit	LI-COR	926-32211
Revert 700 total protein stain	LI-COR	926-11010
MES 20× buffer	Bio-Rad	1610789
Dual color ladder	Bio-Rad	1610374
PVDF membrane	Bio-Rad	1620177
SIRT6 Inhibitor (OSS_128167)	Med Chem Express	HY-107454
PARP1 Inhibitor (Olaparib)	Selleckchem	AZD2281
ADP-HPD	Millipore	118415-60UG
Salmon Sperm DNA	Thermo Fisher	15632-011
Hydroxylamine Hydrochloride	Oakwood Chemical	0999754
HEK 293T cells	ATCC	CRL-11268
HepG2 cells	ATCC	HB-8065
Dulbecco's Modified Eagle Medium (DMEM)	Gibco	11995-065
Opti-MEM	Gibco	31985-070
Fetal bovine serum (FBS)	Gibco	A56708-01
Penicillin-Streptomycin	Gibco	15070-063
Trypsin-EDTA	Gibco	25200-056
Lipofectamine 3000 Transfection Reagent	Thermo Fisher	L3000008

Abbreviations: ATCC, American Type Culture Collection; PVDF, polyvinylidene fluoride.

a The #83732 mAb is discontinued and has been replaced by #89190.

### Cloning

Q5 high-fidelity DNA polymerase (NEB) was used for all PCR amplification steps. All primers were obtained from the DNA/peptide Core at the University of Utah. PCR products were treated with Dpn1 (NEB), and linear products were purified *via* a 1% agarose gel, extracted with QG buffer (Qiagen), and purified by spin column (Qiagen). NEBuilder HiFi DNA Assembly Master Mix (NEB) was used as described by the manufacturer. For blunt-end ligations, T4 PNK (NEB) and T4 DNA ligase (NEB) were used as indicated by the manufacturer. Reactions were transformed into DH5α Mach1 cells (Invitrogen) and plated on antibiotic-containing agar to select colonies for sequence verification. Plasmids were isolated from liquid cultures using Qiagen Miniprep buffers and spin columns as directed by the manufacturer. All plasmids were sequence verified by GENEWIZ (Aventa Life Sciences). Amino acid sequences are listed in the Supporting information. Bacmids were prepared according to the MultiBac protocol from Geneva Biotech.

The Sirt6 C-terminal extension was added to the Sirt6 gene in the plasmid #41565 (pET28a-LIC vector) to generate the full-length Sirt6 gene (aa 1–355). All Sirt6 mutants were then subcloned from this template. PARP1(2-655) was subcloned from the pACeBac1-PARP1 (full-length) plasmid into the pET28a-LIC vector. The pFastBac1 Flag Baf170 #1955 was used as is for protein expression. The MeCP2 and NLK GeneBlocks were assembled into the pET28a-LIC and pACeBac1 vectors, respectively.

### Protein expression

#### E. coli

Plasmids were transformed into BL21 Rosetta(DE3) cells (Millipore) and grown at 37  °C in LB Miller (Thermo Fisher Scientific) to absorbance (600 nm) = 0.6 with shaking at 180 rpm with the appropriate antibiotic. After reaching an absorbance (600 nm) = 0.6 the temperature was changed to 18  °C, and cells were induced with 0.5 mM IPTG and grown for 18 h before harvesting and storage at −80 °C. SIRT6, MeCP2, PARP1(2-655) ARH3, and histones were grown in *E. coli.*

#### Sf9

Bacmids were transfected into Sf9 cells to produce baculovirus and the desired protein as previously described (57). BAF170 and NLK were grown in Sf9 cells.

### Protein purification

Coomassie-stained SDS-PAGE gels are shown in Fig. S1 for the purified proteins.

#### MeCP2

Cell pellets were thawed on ice for 1 h followed by resuspension in lysis buffer (50 mM Tris pH 8.0, 500 mM NaCl, 2 mM MgCl_2_, 5 mM DTT, 1 mM PMSF). Cells were lysed *via* sonication and clarified by centrifugation at 20,000*g* for 30 min. Soluble lysate was applied to equilibrated glutathione resin from GenScript (5 ml of resin per liter of growth) and allowed to batch bind with nutation at room temperature (RT) for 2 h. The resin was washed with 15 column volumes of wash buffer (50 mM Tris pH 8.0, 500 mM NaCl, 2 mM MgCl_2_, 2 mM DTT) and eluted with 30 ml of elution buffer (50 mM Tris pH 8.0, 500 mM NaCl, 2 mM MgCl_2_, 2 mM DTT, 10 mM reduced glutathione). Proteins were either dialyzed overnight against storage buffer or injected on a HiLoad Superdex 16/600 200 pg column (Cytiva) for elution in storage buffer (storage buffer: 50 mM Tris pH 8.0, 100 mM NaCl, 10% glycerol, 2 mM DTT). The fractions were pooled, and protein was concentrated down to the desired concentration as determined by absorbance at 280 nm and an extinction coefficient calculated by ProtParam (https://web.expasy.org/protparam/) and stored at −80 °C.

Note: Addition of 5 mM DTT in the lysis/binding buffer with the addition of excess of glutathione-*S*-transferase resin and longer binding at RT allowed for a better protein yield.

#### Proteins cleaved with ULP1 (SIRT6 WT, PARP1(2-655), ARH3)

Cell pellets were thawed on ice for 1 h followed by resuspension in lysis buffer (40 mM Tris, 500 mM NaCl, 10 mM MgCl_2_, 5 mM β-mercaptoethanol, and 1 mM PMSF, pH 7.5 for SIRT6, 8.0 for PARP1 (2-655)). Lysis buffer for PARP1 (2-655) was supplemented with Protease Inhibitor Mini Tablets (Pierce). Cells were lysed *via* sonication and clarified by centrifugation at 20,000*g* for 30 min. Soluble lysate was applied to lysis buffer-equilibrated nickel resin (GenScript) and allowed to batch bind with nutation at 4 °C for 1 h. The resin was washed with 100 ml lysis buffer per liter of growth supplemented with 30 mM imidazole. Proteins were eluted using 25 ml of lysis buffer supplemented with 300 mM imidazole. Then 25 μl of Ulp1 was added to the elution. The solution was transferred to dialysis tubing (3.5 k molecular weight cutoff, Thermo Fisher Scientific) and allowed to dialyze overnight against lysis buffer containing no imidazole or PMSF at 4 °C with stirring. The next morning, the buffer was replaced with fresh dialysis buffer and allowed 1 h of dialysis. The solution was removed from the dialysis tubing and filtered through 0.45 μm filter to remove precipitation before being applied to equilibrated nickel resin to bind the Ulp1 and SUMO tags (reverse nickel). The solution was nutated with nickel resin for 15 min at 4 °C. The flow-through was obtained and concentrated down to 2 ml for injection on a HiLoad 16/600 Superdex 200 pg (Cytiva) and eluted in storage buffer (50 mM Tris, 100 mM NaCl, 1 mM MgCl_2_,10% glycerol, 2 mM DTT, pH 7.5 for SIRT6 and ARH3 and 8.0 for PARP1 2-655). The fractions were pooled and concentrated down to the desired concentration as determined by absorbance at 280 nm and extinction coefficient calculated by ProtParam (https://web.expasy.org/protparam/) and stored at −80 °C.

Note: The reverse nickel resin volumes and incubation times need to be empirically determined. The cleaved SIRT6 appears to nonspecifically bind the nickel resin so too much resin and/or time will decrease yield, whereas too little resin/time will lead to incomplete removal of the Ulp1 and SUMO.

#### TEV cleavage (SIRT6-H133Y and SIRT6-ΔC298)

The same method was used as for the Ulp1 cleavage except 25 μl of TEV protease was added.

Note: We do not suggest using TEV to cleave SIRT6. In our experience, around 95% of the SIRT6 crashed out during the TEV cleavage and reverse nickel, and there was incomplete cleavage. Also, the SEC cannot separate cleaved *versus* uncleaved SIRT6, leading to a mixture of both proteins. The purification of the SIRT6 with SUMO/Ulp1 worked much better for us.

#### Nickel purification with no cleavage (6XHis SIRT6 WT and 6XHis PARP1 2-655)

Cell pellets were thawed on ice for 1 h followed by resuspension in lysis buffer (40 mM Tris, 500 mM NaCl, 10 mM MgCl_2_, 5 mM β-mercaptoethanol, and 1 mM PMSF, pH 7.5 for SIRT6, 8.0 for PARP1 (2-655)). Lysis buffer for PARP1 1-655 was also supplemented with Protease Inhibitor Mini Tablets (Pierce). Cells were lysed *via* sonication and clarified by centrifugation at 20,000*g* for 30 min. Soluble lysate was applied to equilibrated nickel resin and allowed to batch bind with nutation at 4 °C for 1 h. The resin was washed with 100 ml lysis buffer per liter of growth supplemented with 30 mM imidazole. Proteins were eluted using 25 ml of lysis buffer supplemented with 300 mM imidazole. Eluant was concentrated down to about 2 ml for injection on the HiLoad 16/600 Superdex 200 pg (Cytiva) and eluted in storage buffer (50 mM Tris, 100 mM NaCl, 1 mM MgCl_2_, 10% glycerol, 2 mM DTT, pH 7.5 for SIRT6 and 8.0 for PARP1 2-655). The fractions were pooled and concentrated down to the desired concentration as determined by absorbance at 280 nm and extinction coefficient calculated by ProtParam (https://web.expasy.org/protparam/) and stored at −80 °C.

#### FLAG purifications

BAF170-WT, BAF170-4XHtoG, NLK WT, and NLK Δ(2-54) H(124/125/126)toA were all FLAG purified according to the protocol below (adapted from (75)).

*Buffer A:* 20 mM Hepes, 10 mM KCl, 0.1 mM EDTA, 0.1 mM EGTA, 1 mM DTT, Pierce Protease Inhibitor Mini Tablets (1 per 10 ml), and 0.5 mM PMSF.

*Buffer B:* 20 mM Hepes, 400 mM KCl, 1 mM EDTA, 1 mM EGTA, 10% glycerol, 1 mM DTT, Pierce Protease Inhibitor Mini Tablets (1 per 10 ml), and 0.5 mM PMSF.

*Buffer BC 0*: 20 mM Hepes, 0.2 mM EDTA, 10% glycerol, 1 mM DTT, and 0.2 mM PMSF.

*Buffer BC 100*: 20 mM Hepes, 100 mM KCl, 0.2 mM EDTA, 10% glycerol, 1 mM DTT, and 0.2 mM PMSF.

*Buffer BC 300*: 20 mM Hepes, 300 mM KCl, 0.2 mM EDTA, 10% glycerol, 1 mM DTT, and 0.2 mM PMSF.

*BAF170 WT and 4XHtoG storage buffer:* 20 mM Hepes pH 7.0, 100 mM KCL, 10% glycerol, and 1 mM DTT.

*NLK WT and NLK Δ(2–54) H(124/125/126)toA storage buffer*: 20 mM Hepes pH 7.5, 100 mM KCL, 10% glycerol, and 1 mM DTT.

Buffers were prepared at pH 7.0 for BAF170 constructs and at pH 7.5 for NLK constructs. Purifications were from 1 l of growth from Sf9 cells, stored at −80 °C prior to use. Cell pellets were thawed on ice for 1 h followed by resuspension in 10 ml of buffer A and allowed to swell on ice for 15 min 625 μl of 10% IGEPAL CA-630 per 10 ml of buffer A was added, and the cells were vortexed briefly for lysis. Nuclei were pelleted at 4 °C for 30 s at 17,000*g* and the supernatant was removed. The nuclei were washed once with 10 ml of buffer A and were pelleted at 4 °C for 30 s at 17,000*g*. The resulting nuclear pellet was resuspended in 10 ml of cold nuclear extraction buffer B. Nuclear pellets were then incubated with nutation for 15 min at 4 °C. The supernatant (nuclear extract) was removed and diluted 2-fold with buffer BC-0. This solution was pelleted at 4 °C for 10 min at 17,000*g* to remove precipitation. Soluble nuclear extract was then applied to 1 ml of FLAG resin (GenScript) and incubated for 1 h at 4 °C with nutation. FLAG beads were washed once with 10 bed volumes of buffer BC-100, once with 10 bed volumes of buffer BC-300, and once more with 10 bed volumes of buffer BC-100. The protein of interest was eluted by 3 successive 1-ml washes with buffer BC-100 supplemented with 0.25 mg/ml FLAG peptide (GenScript) for 20 min at 4 °C with nutation. Elutions were combined and either dialyzed overnight against storage buffer or injected on the HiLoad Superdex 16/600,200 pg (Cytiva) and eluted in storage buffer. The proteins were concentrated down to the desired concentration as determined by absorbance at 280 nm and extinction coefficient calculated by ProtParam (https://web.expasy.org/protparam/) and stored at −80 °C.

#### Histones

The histones (full-length H3, H4, H2A, and H2B and 6xHis-SUMO-H3A15C) were purified as described previously (57).

### Peptide and histone semisynthesis

The H3(1–14)K9ac-MESNa thioester, H3(5–13)K9ac-amide, and polyHis peptides (sequences below) were synthesized by solid-phase peptide synthesis and purified by RP-HPLC (Waters C18 column) as described previously (57). The thioester peptide was synthesized on a hydroxy-trityl resin (ChemMatrix) functionalized with hydrazine. The amide-terminated peptides were synthesized on a Rink amide resin (ChemMatrix).

The H3K9ac histone was assembled by native chemical ligation between the H3(1–14)K9ac-MESNa thioester peptide and the H3A15C protein followed by desulfurization and RP-HPLC purification as described previously (57). The HPLC and mass spectrometric characterization of the three peptides and the ligated, desulfurized histone are shown in Figs. S5 and S6*A*. The RP-HPLC characterization was performed on an Agilent 1260 Infinity HPLC system using a Waters C18 column (#186003624) in a water/acetonitrile/0.1% TFA solvent system. The mass spectrometry analysis was performed on a Waters Acuity QDa.

H3(1-14)K9ac-MESNa thioester: ARTKQTAR(Kac)STGGK-C=OS(MESNa)

H3(5-13)K9ac-amide: Ac-QTAR(Kac)STGG-NH_2_

polyHis peptide: HHHHHHGGG-NH_2_

### Octamer and mononucleosome preparation

H3WT and H3K9ac octamers were assembled by dialysis and purified by size-exclusion chromatography (Superdex 200 increase column, Cytiva) as described previously (57). The mononucleosomes were prepared by mixing the respective octamer with 177 bp ds601 10.13039/100026054DNA (sequence in Supporting information) and dialyzing as described previously (57). The mononucleosomes were analyzed on a native 5% Tris-borate-EDTA (TBE) gel to assess their quality (Fig. S6).

### Cell culture

HEK293T and HepG2 were purchased directly from American Type Culture Collection. The cells were grown in Dulbecco's modified Eagle medium (DMEM) with supplementation of 10% fetal bovine serum and 1% penicillin/streptomycin, under 5% CO2. Cells were split at 1:10 after reaching 95% confluency *via* trypsin.

### Cell transfections

Ten centimeter plates were seeded so that cells reached 80% confluency at time of transfection. Cells were transfected in the evening with 5 μg of each plasmid (10 μg total for the set of HA-YY1 and FLAG-SIRT6) per 10-cm plate. Cells were transfected according to manufacturer guidelines with 20 μl of lipofectamine 3000 and 20 μl of P3000 reagent per 10-cm plate in 5 ml of Opti-MEM. Cell media was replaced the next morning with 10 ml DMEM supplemented with 10% fetal bovine serum but no antibiotics. Cells were harvested by scraping and centrifugation in cold 1× PBS 24 h after being placed in DMEM followed by immediate lysis for immunoprecipitation.

### Immunoprecipitation

Cells (HEK293T or HepG2) were lysed in 100 μl of lysis buffer per 10-cm plate (50 mM Tris pH 7.5, 150 mM NaCl, 1% NP-40, 1× complete protease inhibitor (EDTA free), 10 μM olaparib, 1 μM ADP-HPD) on ice for 10 min on ice followed by sonication at 15% amplitude for 10 s with two reps. Lysate was clarified by centrifugation at 17,000*g* for 10 min at 4 °C. 10% of the soluble fraction was saved for input analysis and the rest was applied to anti-HA magnetic beads (12.5 μl of beads per 100 μl of lysis) and nutated at RT for 1 h. Anti-HA magnetic beads were washed 3 × 200 μl (per 12.5 μl beads) with lysis buffer (without olaparib or ADP-HPD supplementation) and eluted with 20 μl of 2.5× SDS-loading buffer (per 12.5 μl HA beads) at 95 °C for 5 min. Western blot analysis followed with 10% input loading and immunoprecipitation samples from 1× 10-cm plate. Membranes were initially probed for anti-YY1 (1:142) and anti-mono-ADPr (1:1000). Membranes were successively probed for anti-FLAG (1:2000) for detection of transfected FLAG-SIRT6 in the inputs.

### Western blotting

Reactions were loaded on a 4 to 12% bis tris gel and run with Bio-Rad MES buffer at 180 V for about 45 min. We note that some of the gels were cast by us and others were purchased precast from Bio-Rad, giving some variation in bandwidth and appearance. Gels were transferred to Bio-Rad polyvinylidene fluoride membrane with cold towbin buffer (25 mM Tris, 192 mM glycine, 10% (v/v) methanol, 0.06% (w/v) SDS) using a semidry transblot turbo set (Bio-Rad) for 30 min at 20 V. The membranes with total protein quantification were subjected to Revert Total Protein Stain 700 (Licor) according to the manufacturer’s protocol. All membranes were blocked with 3% nonfat milk (Genesee) in Tris-buffered saline with Tween 20 (TBST) (50 mM Tris pH 7.5, 150 mM NaCl, 0.05% Tween-20) at RT for 1 h. Anti-ADPr blots were performed using a 1:1000 antibody dilution in TBST of the Cell Signaling Technologies pan/mono antibody (#83732) overnight at 4 °C. The blot in Fig. S1 was incubated with a 1:1000 dilution in TBST of the Anti-ADPr Millipore binding reagent overnight at 4 °C. Anti-H3K9ac and anti-H3 (Cell Signaling Technologies) were used at a 1:2000 dilution. Anti-SIRT6 and anti-His tag (Cell Signaling Technologies) were used at a 1:1000 dilution. The blots were imaged on a Licor Odyssey system.

The specificity of the ADP-ribose antibodies was shown by using no cofactor (*i.e.*, NAD^+^) and/or no enzyme lanes in the assays and by agreement between the two different antibodies (*i.e.*, Figs. 2*A* and S2*A*). The H3K9ac antibody was similarly validated by using a “no SIRT6” condition along with the other conditions tested (Fig. S3*C*) on semisynthetically generated H3K9ac nucleosomes. The SIRT6 and H3 antibodies were assessed by the band aligning with the correct molecular weight for the respective recombinant protein as observed in a total protein stain. The His tag antibody was assessed by blotting for the SIRT6 before and after removal of the 6xHis tag by proteolytic cleavage.

### ADP-ribosylation assays

The reactions (15 μl total volume) were set up and incubated as described in the respective figure legends. NAD^+^ was added last to initiate the reaction. A 5× reaction buffer was diluted to 1× for the assay. The 1× reaction buffer was 50 mM Tris pH 7.5, 20 mM NaCl, 2 mM MgCl_2_, 2 mM DTT, and 1 mM NAD^+^ (NEB) was used when not otherwise specified in figure legends. The reactions were quenched by addition of 6× SDS loading buffer and boiled for 5 min.

Note: Reactions with SIRT6 and PARP1 (2-655) contained 10 μm SIRT6 and 2 μm PARP1 (2-655) to observe SIRT6 modification of PARP1 (2-655).

Note: SIRT6 auto mARylation reactions were set up (2 μm SIRT6) with no other substrate.

### Electrophoretic mobility shift assays

The SIRT6-WT or SIRT6-ΔC were incubated with 50 nM of ds601 DNA in reaction buffer (50 mM Tris pH 7.5, 20 mM NaCl, 2 mM MgCl_2_, 2 mM DTT) at 10 μl total volume. The reactions were incubated at RT for 15 min followed by the addition of 6× purple DNA loading dye (NEB). The reactions were analyzed on a 5% TBE gel with cold 1× TBE buffer (89 mM Tris, 89 mM boric acid, 2 mM EDTA) for 100 min at 120 V, stained with ethidium bromide, and imaged using a Bio-Rad ChemiDoc system.

### HPLC deacetylation assays

The reactions (50 μl total volume) included 10 μm 6xHis-SIRT6, 600 μm H3K9ac peptide, 1 mM NAD^+^ in reaction buffer (50 mM Tris pH 7.5, 20 mM NaCl, 2 mM MgCl_2_, 2 mM DTT) and were incubated at 30 °C for 1 h. The reactions were quenched by the addition of 50 μl solvent A (water, 0.1% TFA). The samples were analyzed on a C18 column (Waters #186003624) using a gradient of 0 to 20% solvent B over 20 min (solvent B = 90% acetonitrile, 10% water, 0.1% TFA). After 20 min, the column washed with 100% solvent B for 5 min, and then re-equilibrated in 100% solvent A for 5 min. An Agilent 1260 Infinity system was used.

### H3K9ac mononucleosome deacetylation assay

The reactions (15 μl total volume) included 2 μm 6xHis-SIRT6 and 150 nM H3K9ac mononucleosome in reaction buffer (50 mM Tris pH 7.5, 20 mM NaCl, 2 mM MgCl_2_, 2 mM DTT, 1 mM NAD^+^) and were incubated at 30 °C for 10 min. The reactions were quenched with 6× SDS loading buffer and boiled for 5 min.

### Thrombin cleavage assay

The SIRT6 auto-ADP-ribosylation assay was set up as before with incubation for 20 min at 37  °C. To the reactions was added 1 U of thrombin (Cytiva) reconstituted in 1× PBS at 1 U/μl. The reactions were incubated for an additional 20 min at 37  °C and then quenched with 6× SDS loading buffer.

### ARH3 assay

MeCP2 mARylation assay was set up as previously described. Reactions were quenched with 1 mM SIRT6 inhibitor (MedChem Express, OSS_128167) after 2 h and additionally incubated for either 30 min or 2 h more at 30  °C with 1 μM ARH3. We note that the SIRT6 inhibitor was dissolved to 100 mM in 100% dimethylsulfoxide and a working stock of 10 mM in 90% water and 10% dimethylsulfoxide was diluted from that. This 10 mM stock was cloudy and was homogenized thoroughly before adding to the reactions. Fig. S3*E* shows the inhibition of SIRT6 by OSS_128167 (*e.g.*, lane 2 compared to lane 3).

### Hydroxylamine treatment

Hydroxylamine hydrochloride was dissolved in water at 5 M and adjusted to pH 7.0. The standard MeCP2 WT mARylation reaction was performed, 5× SDS-LB was added, and samples were boiled for 5 min. Then, the hydroxylamine solution was added to bring the final concentration to 0.5 or 1.0 M, and the reactions were incubated for 2 h at RT. Reactions were immediately run for Western blot analysis.

### Mass spectrometric analysis of ADP-ribosylated MeCP2

A single 100-μl reaction of MeCP2 (10 μM), SIRT6 (10 μM), ds601 (1 μM), and NAD^+^ (1 mM) in buffer (50 mM Tris pH 7.5, 20 mM NaCl, 2 mM MgCl_2_, 2 mM DTT) was incubated at 37 °C for 2 h, and then SDS-loading buffer was added to the reaction. The mass spectrometric analysis was carried out by Creative Proteomics as described in Data File S3. The full results are tabulated in Data File S2.

### Software and data analysis

Prism GraphPad (https://www.graphpad.com) was used for preparing the plots in Figures 1*A*, S3*D*, S5, and S6. The unpaired, two-tailed *t* test in Figure 1*A* was performed in Prism GraphPad. The exact *p* values are shown in the figure legends. The densitometric analyses was performed in Licor Image Studio with normalization to a loading control band and then to a control condition (control = 1.0). All tabulated values for the densitometry measurements and subsequent calculations are in Data File S4. The final normalized values are displayed in the respective blots (“Quant:”). The uncropped blots and gels, including all experimental replicates are in Data File S5. The number of replicates for each experiment is noted in the figure legends. The sequence alignments were performed using NCBI Protein BLAST by searching in the UniProtKB/Swiss-Prot database.

## Data availability

All the data from this study are contained within this article. The mass spectrometry proteomics data have been deposited to the ProteomeXchange Consortium *via* the PRIDE partner repository with the dataset identifier PXD061933.

## Supporting information

This article contains supporting information.

## Conflict of interest

The authors declare that they have no conflicts of interest with the contents of this article.
