# Supplementary material for: DNA stimulates the deacetylase SIRT6 to mono-ADP-ribosylate proteins with histidine repeats
Source: J Biol Chem. 2025 Apr 23;301(6):108532. doi: 10.1016/j.jbc.2025.108532 (PMC12167490; doi:10.1016/j.jbc.2025.108532)
Supplement: Supporting Information [file mmc1.pdf]

Supporting Information for

DNA stimulates the deacetylase SIRT6 to mono-ADP-ribosylate proteins with histidine repeats

Nicholas J. Pederson[1] and Katharine L. Diehl[1*]

1 Department of Medicinal Chemistry, University of Utah
*correspondence to k.diehl@utah.edu

Contents:

Supporting Figures S1-S6
Annotated amino acid sequences for all proteins
Sequences for the DNA used in the enzyme assays

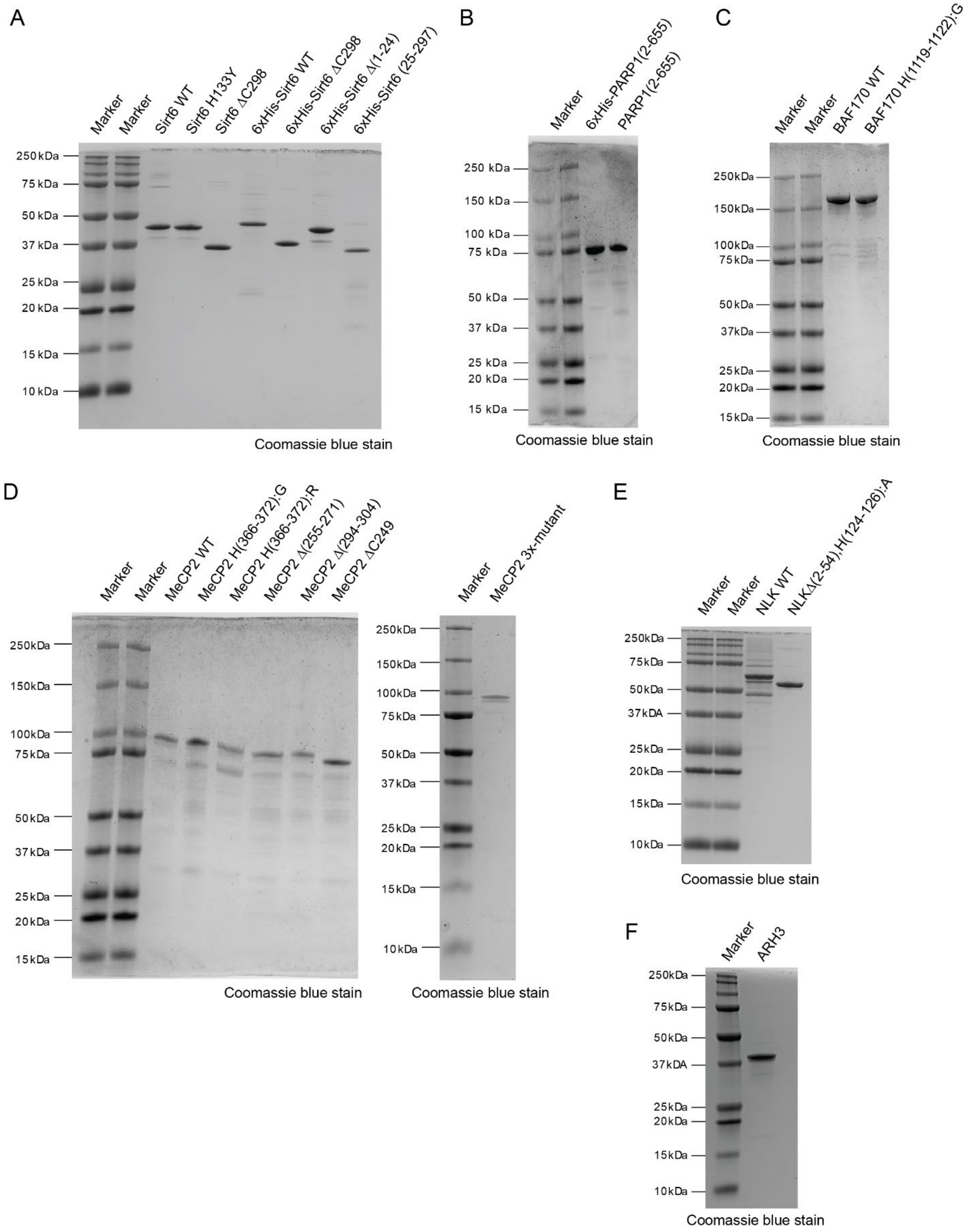

Figure S1. Gel analysis of proteins used in this study. A) SIRT6, B) PARP1, C) BAF170/SMARCC2, D) MeCP2, E) NLK, F) ARH3, n = 1.

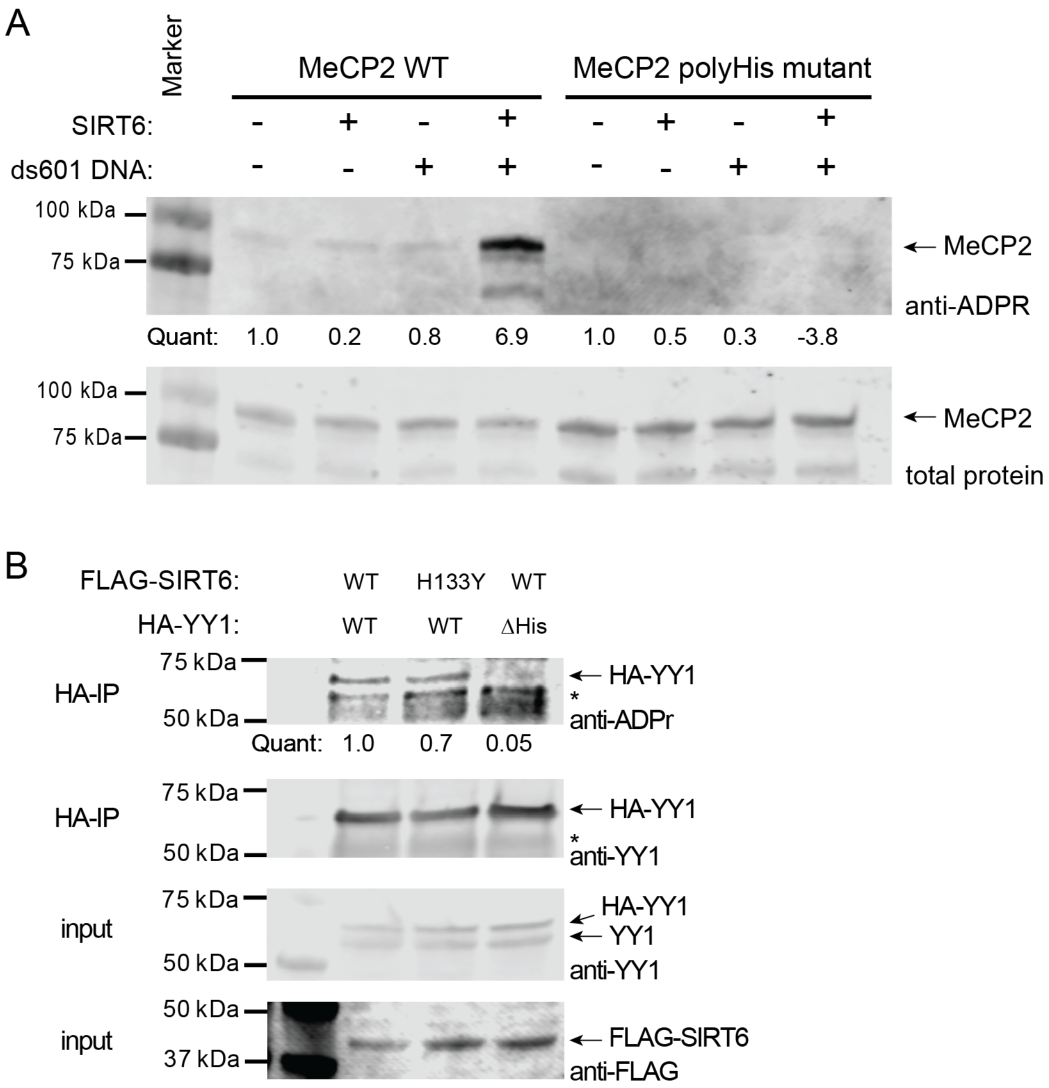

Figure S2. Supporting data related to Figure 2. A) Immunoblot analysis of MeCP2-WT or -polyHis mutant (2 μM) mARylation by SIRT6 WT (2 μM) in the presence of ds601 DNA (1 μM) at 37°C for 2 h Western Blot developed with 1:1000 dilution of the Millipore pan/mono ADPR binding reagent (#MABE1016), n = 2. B) Immunoblot analysis of immunoprecipitated HA-YY1-WT or HA-YY1-ΔpolyHis from HepG2 cells expressing the respective YY1 protein and either FLAG-SIRT6-WT or FLAG-SIRT6-H133Y. The anti-ADPr blot was obtained used a mono-ADP-ribose binding reagent (Millipore #MABE1076), n = 1.

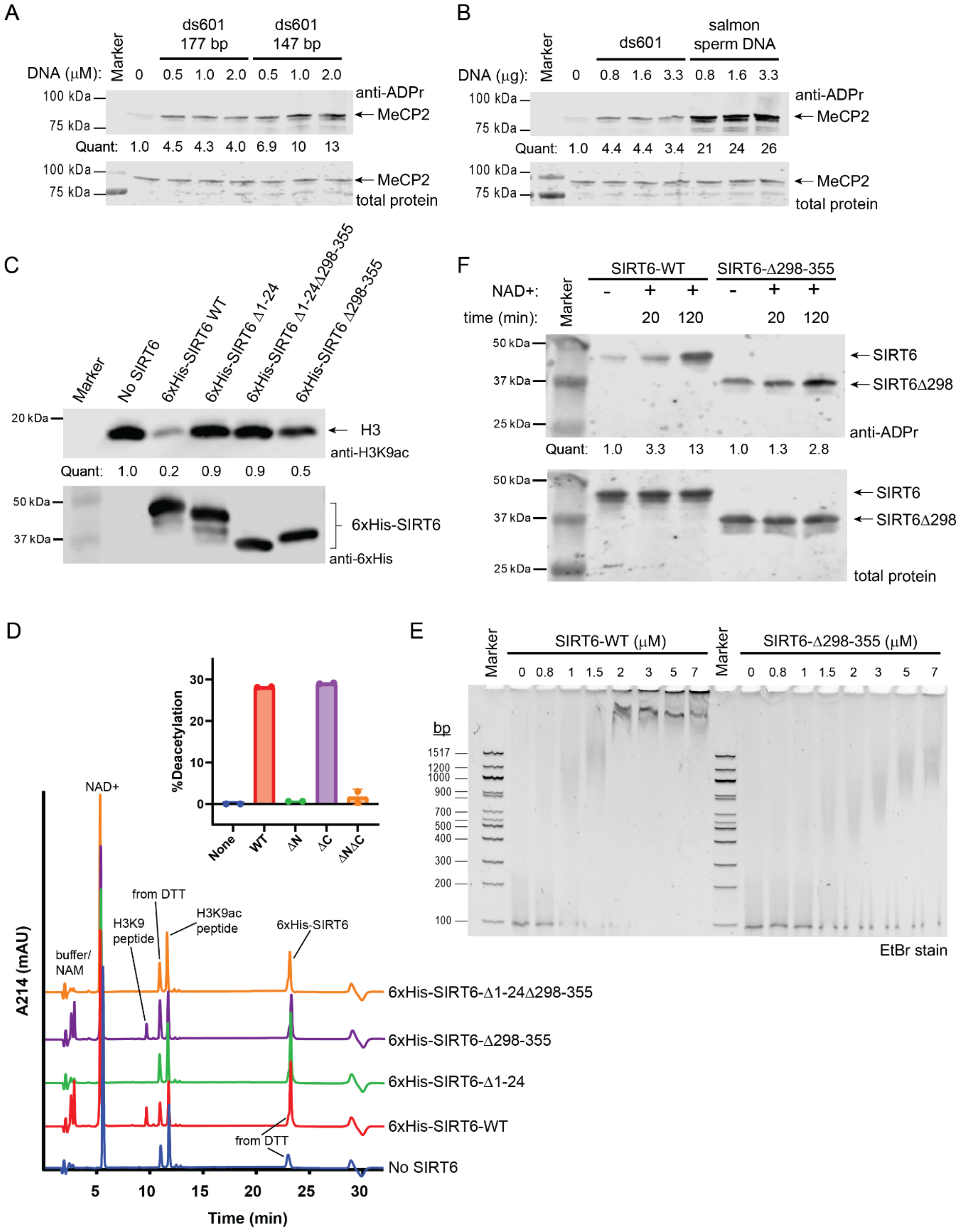

Figure S3. Supporting data related to Figure 3. A) Titration of ds601 177 bp or 147 bp in the MeCP2 (2 μM) mARylation assay by SIRT6 (2 μM), n = 2. B) Titration of ds601 177 bp or salmon sperm DNA in the MeCP2 (2 μM) mARylation assay by SIRT6 (2 μM), n = 2. C) Immunoblot analysis of H3K9ac nucleosome (150 nM) deacetylation assays with 6xHis-SIRT6 mutants (2 μM) at 30°C for 20 min, n = 2. D) Chromatograms of H3K9ac peptide (600 μM) assays with 6xHis-SIRT6 (10

μM),1 mM NAD^+, at 37°C for 1 h, λ = 214 nm, n = 2. The inset shows the extent of deacetylation that was calculated by quantifying the area under the deacetylated peptide peak and normalizing to the SIRT6 peak. The error bars represent ± S.D. E) Electrophoretic mobility shift assay (EMSA) of ds601 (100 nM) with Cleaved-SIRT6-WT or -ΔC at the indicated concentrations (in μM). The TBE gel was stained with EtBr, n = 3. F) Immunoblot analysis of autoMARylation by SIRT6-WT or -ΔC (2 μM) and 1 mM NAD^+, n = 2.

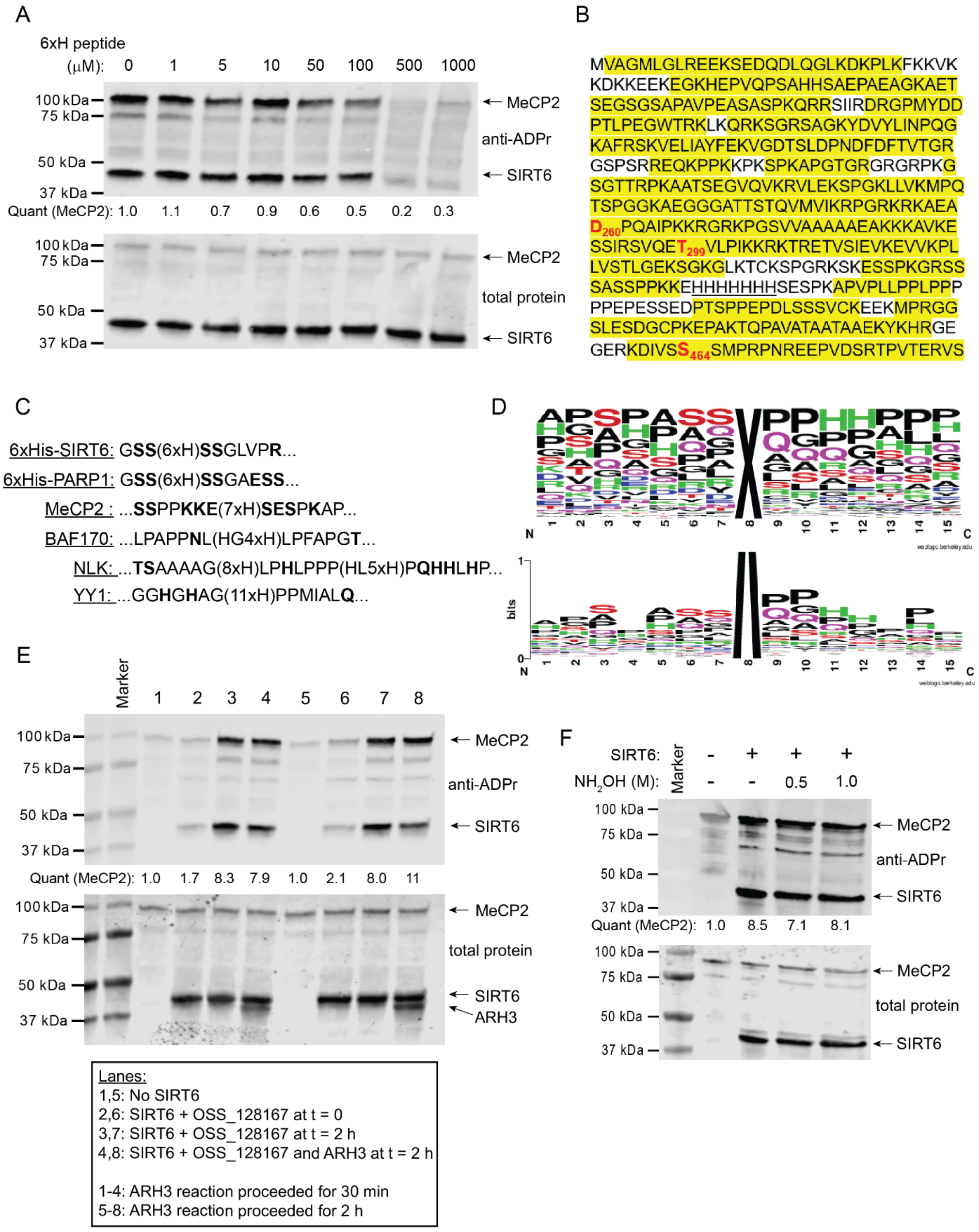

Figure S4. Supporting data related to Figure 4. A) Immunoblot analysis of MeCP2 (2 μM) and SIRT6 (2 μM) mARylation reactions (1 μM ds601, 1 mM NAD^+, 37°C for 2 h) with the 6xH peptide (HHHHHHGGG), n = 2. B) Sequence of MeCP2. The yellow-highlighted portions were observed in the analysis by the identification of at least one peptide containing those amino

acids (85% sequence coverage for this 486-amino acid protein), while the non-highlighted amino acids were not observed. The amino acids in red were found to be mARylated. The polyHis tract is underlined. All peptides that were identified are listed in **Data File S2**. C) Sequences surrounding the polyHis tract in the proteins tested in this study. Any residues that could be mARylation acceptors are bolded. D) Frequency map (top) and sequence map (bottom) for the surrounding residues of the polyHis tracts in all 129 of human polyHis proteins (listed in **Data File S1**). Position 8 ("X") represents the polyHis tract, which was defined as at least four sequential histidine residues plus histidines that were separated from the sequential histidine tract by no more than one intervening amino acid (e.g., HGHHHH or HHHHHSHRHH). E) Immunoblot analysis of MeCP2 (2 µM) and SIRT6 (2 µM) mARylation reactions (1 µM ds601, 1 mM NAD^+, 37°C for 2 h). After the 2 h mARylation reaction, SIRT6 inhibitor (OSS_128167, 1 mM) was added to quench the SIRT6. At the same time, ARH3 (1 µM) was added to reactions 7 and 8. Reactions 1-4 were then allowed to proceed for an additional 30 min. Reactions 5-8 were allowed to proceed for an additional 2 h, n = 2. F) Immunoblot analysis of MeCP2 (2 µM) and SIRT6 (2 µM) mARylation reactions (1 µM ds601, 1 mM NAD^+, 37°C for 2 h). The samples in the indicated lanes were treated with 0.5 or 1 M hydroxylamine (NH_2OH) for 2 h at RT, n = 2.

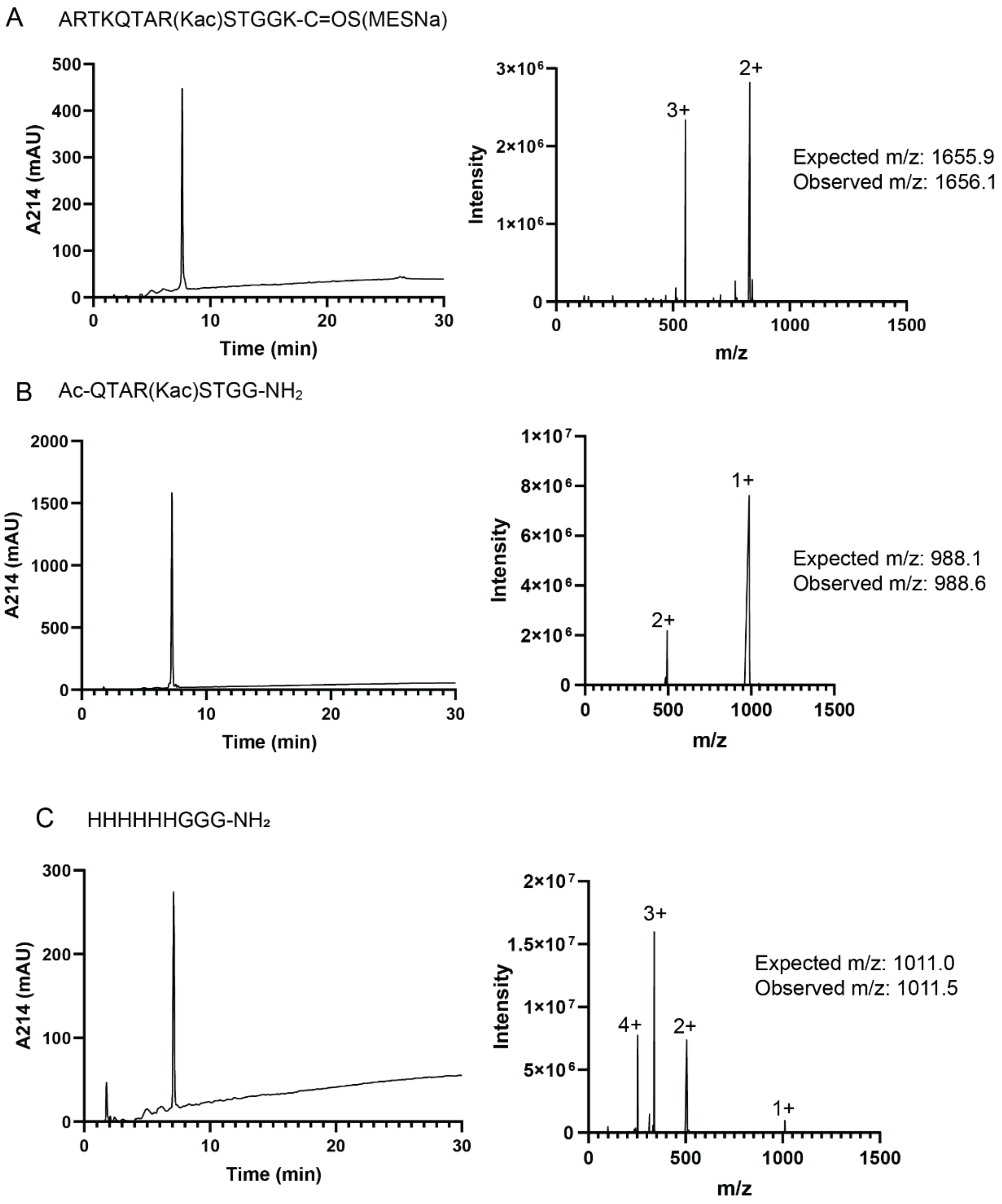

Figure S5. Chromatographic and mass spectrometric analysis of the peptides used in this study. A) H3(1-14)K9ac-MESNa thioester: ARTKQTAR(Kac)STGGK-COS(MESNa), B) H3(5-13)K9ac-amide: Ac-QTAR(Kac)STGG-NH_2, C) polyHis peptide: HHHHHHGGG-NH_2. The chromatograms (left) were collected using a 0-70% solvent B gradient over 30 min (solvent A = 100% water, 0.1% TFA and solvent B = 90% water, 10% water, 0.1% TFA) on a Waters BEH XBridge column (186003624) with detection of absorbance of 214 nm. The peak at 2 min is the void volume. The extracted ion chromatograms (right) were collected using a Waters QDa.

A

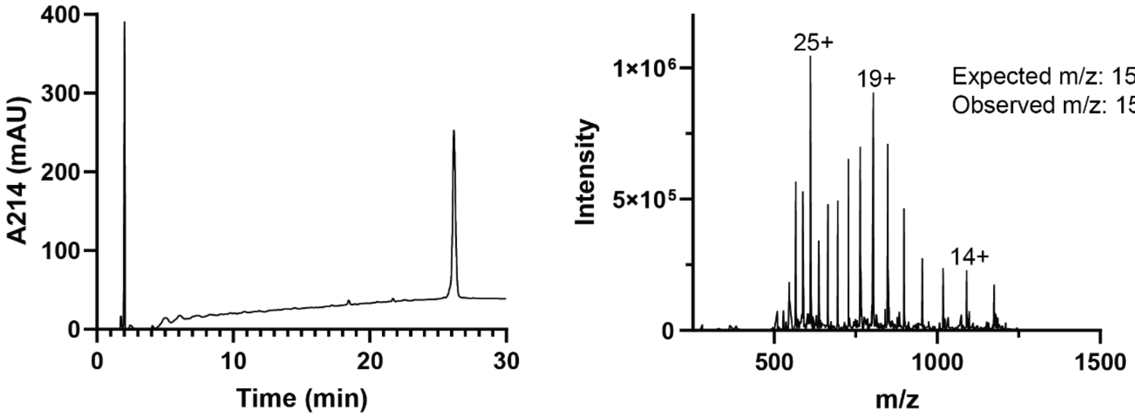

B

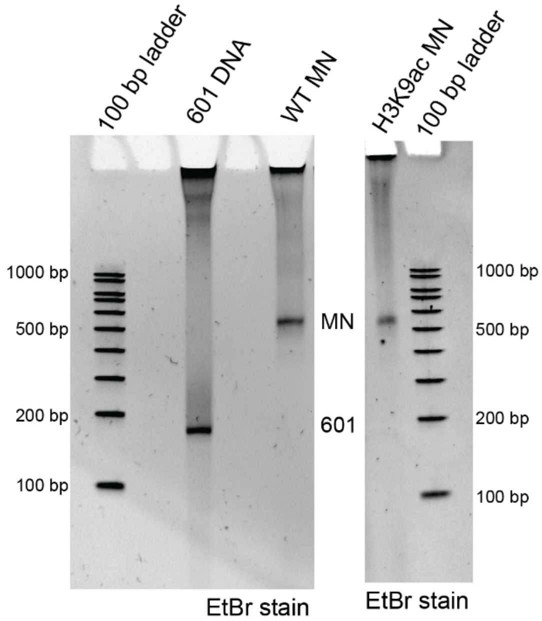

Figure S6. Analysis of the H3K9ac histone and WT and H3K9ac mononucleosomes. A) UV and extracted ion chromatograms for the H3K9ac histone. This sample was analyzed as described in the **Figure S5** legend. B) Native gel analysis of the WT (left) and H3K9ac (right) mononucleosomes ("MN") and the 177-bp 601 dsDNA (left) (5% TBE gel, EtBr staining), n = 1. Note that Apex 100 bp ladder (Genesee #19-109) was used for these gels.

Protein Sequences:

PARP1:

6XHis-PARP1 (2-655): Highlight = 6XHis Tag, in pet28a plasmid

MGSSHHHHHHSSGAESSDKLYRVEYAKSGRASCKKCSESIPKDSLRMAIMVQSPMFDGKVPH
WYHFSCFWKVGHSIRHPDVEVDGFSELRWDDQQKVKKTAEAGGVTGKGQDGIGSKAEKTLG
DFAAEYAKSNRSTCKGCMEKIEKGQVRLSKKMVDPEKPQLGMIDRWYHPGCFVKNREELGFR
PEYSASQLKGFSLLATEDKEALKKQLPGVKSEGKRKGDEVDGVDEVAKKKSKKEKDKDSKLEK
ALKAQNDLIWNIKDELKKVCSTNDLKELLIFNKQQVPSGESAILDRVADGMVFGALLPCEECSG
QLVFKSDAYYCTGDVTAWTKCMVKTQTPNRKEWVTPKEFREISYLKKLKVKKQDRIFPPETSA
SVAATPPPSTASAPAAVNSSASADKPLSNMKILTLGKLSRNKDEVKAMIEKLGGKLTGTANKAS
LCISTKKEVEKMNKKMEEVKEANIRVVSEDFLQDVSASTKSLQELFLAHILSPWGAEVKAEPVE
VVAPRGKSGAALSKKSKGQVKEEGINKSEKRMKLTLKGGAAVDPDSGLEHSAHVLEKGGKVF
SATLGLVDIVKGTNSYYKLQLLEDDKENRYWIFRSWGRVGTVIGSNKLEQMPSKEDAIEHFMKL
YEEKTGNAWHSKNFTKYPKKFYPLEIDYGQDEEAVKKL

Cleaved PARP1 (2-655): in pet28a plasmid

AESSDKLYRVEYAKSGRASCKKCSESIPKDSLRMAIMVQSPMFDGKVPHWYHFSCFWKVGHS
IRHPDVEVDGFSELRWDDQQKVKKTAEAGGVTGKGQDGIGSKAEKTLGDFAAEYAKSNRSTC
KGCMEKIEKGQVRLSKKMVDPEKPQLGMIDRWYHPGCFVKNREELGFRPEYSASQLKGFSLL
ATEDKEALKKQLPGVKSEGKRKGDEVDGVDEVAKKKSKKEKDKDSKLEKALKAQNDLIWNIKD
ELKKVCSTNDLKELLIFNKQQVPSGESAILDRVADGMVFGALLPCEECSGQLVFKSDAYYCTGD
VTAWTKCMVKTQTPNRKEWVTPKEFREISYLKKLKVKKQDRIFPPETSASVAATPPPSTASAPA
AVNSSASADKPLSNMKILTLGKLSRNKDEVKAMIEKLGGKLTGTANKASLCISTKKEVEKMNKK
MEEVKEANIRVVSEDFLQDVSASTKSLQELFLAHILSPWGAEVKAEPVEVVAPRGKSGAALSKK
SKGQVKEEGINKSEKRMKLTLKGGAAVDPDSGLEHSAHVLEKGGKVFSATLGLVDIVKGTNSY
YKLQLLEDDKENRYWIFRSWGRVGTVIGSNKLEQMPSKEDAIEHFMKLYEEKTGNAWHSKNFT
KYPKKFYPLEIDYGQDEEAVKKL

MeCP2:

MeCP2 WT (2-486): Highlight = GST tag, in pet28a plasmid

MSPILGYWKIKGLVQPTRLLLEYLEEKYEEHLYERDEGDKWRNKKFELGLEFPNLPYYIDGDVK
LTQSMAIIRYIADKHNMLGGCPKERAEISMLEGAVLDIRYGVSRIAYSKDFETLKVDFLSKLPEML
KMFEDRLCHKTYLNGDHVTHPDFMLYDALDVVLYMDPMCLDAFPKLVCFKKRIEAIPQIDKYLK
SSKYIAWPLQGWQATFGGGDHPPKLVPRGSVAGMLGLREEKSEDQDLQGLKDKPLKFKKVKK
DKKEEKEGKHEPVQPSAHHSAEPAEAGKAETSEGSGSAPAVPEASASPKQRRSIIRDRGPMY
DDPTLPEGWTRKLKQRKSGRSAGKYDVYLINPQGKAFRSKVELIAYFEKVGDTSLDPNDFDFT
VTGRGSPSRREQKPPKKPKSPKAPGTGRGRGRPKGSGTTRPKAATSEGVQVKRVLEKSPGK
LLVKMPFQTSPGGKAEGGGATTSTQVMVIKRPGRKRKAEADPQAIPKKRGRKPGSVVAAAAA
EAKKKAVKESSIRSVQETVLPIKKRKTRETVSIEVKEVVKPLLVSTLGEKSGKGLKTCKSPGRKS
KESSPKGRSSSASSPPKKEHHHHHHHSESPKAPVPLLPPLPPPPPEPESSEDPTSPPEPQDLS
SSVCKEEKMPRGGSLESDGCPKEPAKTQPAVATAATAAEKYKHRGEGERKDIVSSSMPRPNR
EEPVDSRTPVTERVS

MeCP2 H (366-372) toG: Underline = HtoG mutation, Highlight= GST tag, in pet28a plasmid

MSPILGYWKIKGLVQPTRLLLEYLEEKYEEHLYERDEGDKWRNKKFELGLEFPNLPYYIDGDVK
LTQSMAIIRYIADKHNMLGGCPKERAEISMLEGAVLDIRYGVSRIAYSKDFETLKVDFLSKLPEML
KMFEDRLCHKTYLNGDHVTHPDFMLYDALDVVLYMDPMCLDAFPKLVCFKKRIEAIPQIDKYLK
SSKYIAWPLQGWQATFGGGDHPPKLVPRGSVAGMLGLREEKSEDQDLQGLKDKPLKFKKVKK
DKKEEKEGKHEPVQPSAHHSAEPAEAGKAETSEGSGSAPAVPEASASPKQRRSIIRDRGPMY
DDPTLPEGWTRKLKQRKSGRSAGKYDVYLINPQGKAFRSKVELIAYFEKVGDTSLDPNDFDFT
VTGRGSPSRREQKPPKKPKSPKAPGTGRGRGRPKGSGTTRPKAATSEGVQVKRVLEKSPGK
LLVKMPFQTSPGGKAEGGGATTSTQVMVIKRPGRKRKAEADPQAIPKKRGRKPGSVVAAAAA
EAKKKAVKESSIRSVQETVLPIKKRKTRETVSIEVKEVVKPLLVSTLGEKSGKGLKTCKSPGRKS
KESSPKGRSSSASSPPKKEGGGGGGGSESPKAPVPLLPPLPPPPPEPESSEDPTSPPEPQDL
SSSVCKEEKMPRGGSLESDGCPKEPAKTQPAVATAATAAEKYKHRGEGERKDIVSSSMPRPN
REEPVDSRTPVTERVS

MeCP2 H(366-372)toR: Underline = HtoR mutation, Highlight= GST tag, in pet28a plasmid

MSPILGYWKIKGLVQPTRLLLEYLEEKYEEHLYERDEGDKWRNKKFELGLEFPNLPYYIDGDVK
LTQSMAIIRYIADKHNMLGGCPKERAEISMLEGAVLDIRYGVSRIAYSKDFETLKVDFLSKLPEML
KMFEDRLCHKTYLNGDHVTHPDFMLYDALDVVLYMDPMCLDAFPKLVCFKKRIEAIPQIDKYLK
SSKYIAWPLQGWQATFGGGDHPPKLVPRGSVAGMLGLREEKSEDQDLQGLKDKPLKFKKVKK
DKKEEKEGKHEPVQPSAHHSAEPAEAGKAETSEGSGSAPAVPEASASPKQRRSIIRDRGPMY
DDPTLPEGWTRKLKQRKSGRSAGKYDVYLINPQGKAFRSKVELIAYFEKVGDTSLDPNDFDFT
VTGRGSPSRREQKPPKKPKSPKAPGTGRGRGRPKGSGTTRPKAATSEGVQVKRVLEKSPGK
LLVKMPFQTSPGGKAEGGGATTSTQVMVIKRPGRKRKAEADPQAIPKKRGRKPGSVVAAAAA
EAKKKAVKESSIRSVQETVLPIKKRKTRETVSIEVKEVVKPLLVSTLGEKSGKGLKTCKSPGRKS
KESSPKGRSSSASSPPKKERRRRRRRSESPKAPVPLLPPLPPPPPEPESSEDPTSPPEPQDLS
SSVCKEEKMPRGGSLESDGCPKEPAKTQPAVATAATAAEKYKHRGEGERKDIVSSSMPRPNR
EEPVDSRTPVTERVS

MeCP2 Δ255-271: Highlight= GST tag, in pet28a plasmid

MSPILGYWKIKGLVQPTRLLLEYLEEKYEEHLYERDEGDKWRNKKFELGLEFPNLPYYIDGDVK
LTQSMAIIRYIADKHNMLGGCPKERAEISMLEGAVLDIRYGVSRIAYSKDFETLKVDFLSKLPEML
KMFEDRLCHKTYLNGDHVTHPDFMLYDALDVVLYMDPMCLDAFPKLVCFKKRIEAIPQIDKYLK
SSKYIAWPLQGWQATFGGGDHPPKLVPRGSVAGMLGLREEKSEDQDLQGLKDKPLKFKKVKK
DKKEEKEGKHEPVQPSAHHSAEPAEAGKAETSEGSGSAPAVPEASASPKQRRSIIRDRGPMY
DDPTLPEGWTRKLKQRKSGRSAGKYDVYLINPQGKAFRSKVELIAYFEKVGDTSLDPNDFDFT
VTGRGSPSRREQKPPKKPKSPKAPGTGRGRGRPKGSGTTRPKAATSEGVQVKRVLEKSPGK
LLVKMPFQTSPGGKAEGGGATTSTQVMVIKRPGRKPGSVVAAAAAEAKKKAVKESSIRSVQET
VLPIKKRKTRETVSIEVKEVVKPLLVSTLGEKSGKGLKTCKSPGRKSKESSPKGRSSSASSPPK
KEHHHHHHHSESPKAPVPLLPPLPPPPPEPESSEDPTSPPEPQDLSSSVCKEEKMPRGGSLES
DGCPKEPAKTQPAVATAATAAEKYKHRGEGERKDIVSSSMPRPNREEPVDSRTPVTERVS

MeCP2 Δ294-304: Highlight= GST tag, in pet28a plasmid

MSPILGYWKIKGLVQPTRLLLEYLEEKYEEHLYERDEGDKWRNKKFELGLEFPNLPYYIDGDVK
LTQSMAIIRYIADKHNMLGGCPKERAEISMLEGAVLDIRYGVSRIAYSKDFETLKVDFLSKLPEML
KMFEDRLCHKTYLNGDHVTHPDFMLYDALDVVLYMDPMCLDAFPKLVCFKKRIEAIPQIDKYLK
SSKYIAWPLQGWQATFGGGDHPPKLVPRGSVAGMLGLREEKSEDQDLQGLKDKPLKFKKVKK
DKKEEKEGKHEPVQPSAHHSAEPAEAGKAETSEGSGSAPAVPEASASPKQRRSIIRDRGPMY
DDPTLPEGWTRKLKQRKSGRSAGKYDVYLINPQGKAFRSKVELIAYFEKVGDTSLDPNDFDFT
VTGRGSPSRREQKPPKKPKSPKAPGTGRGRGRPKGSGTTRPKAATSEGVQVKRVLEKSPGK
LLVKMPFQTSPGGKAEGGGATTSTQVMVIKRPGRKRKAEADPQAIPKKRGRKPGSVVAAAAA
EAKKKAVKESSIKRKTRETVSIEVKEVVKPLLVSTLGEKSGKGLKTCKSPGRKSKESSPKGRSS
SASSPPKKEHHHHHHHSESPKAPVPLLPPLPPPPPEPESSEDPTSPPEPQDLSSSVCKEEKMP
RGGSLESDGCPKEPAKTQPAVATAATAAEKYKHRGEGERKDIVSSSMPRPNREEPVDSRTPV
TERVS

MeCP2 ΔdelC459 (2-458): Highlight= GST tag, in pet28a plasmid

MSPILGYWKIKGLVQPTRLLLEYLEEKYEEHLYERDEGDKWRNKKFELGLEFPNLPYYIDGDVK
LTQSMAIIRYIADKHNMLGGCPKERAEISMLEGAVLDIRYGVSRIAYSKDFETLKVDFLSKLPEML
KMFEDRLCHKTYLNGDHVTHPDFMLYDALDVVLYMDPMCLDAFPKLVCFKKRIEAIPQIDKYLK
SSKYIAWPLQGWQATFGGGDHPPKLVPRGSVAGMLGLREEKSEDQDLQGLKDKPLKFKKVKK
DKKEEKEGKHEPVQPSAHHSAEPAEAGKAETSEGSGSAPAVPEASASPKQRRSIIRDRGPMY
DDPTLPEGWTRKLKQRKSGRSAGKYDVYLINPQGKAFRSKVELIAYFEKVGDTSLDPNDFDFT
VTGRGSPSRREQKPPKKPKSPKAPGTGRGRGRPKGSGTTRPKAATSEGVQVKRVLEKSPGK
LLVKMPFQTSPGGKAEGGGATTSTQVMVIKRPGRKRKAEADPQAIPKKRGRKPGSVVAAAAA
EAKKKAVKESSIRSVQETVLPIKKRKTRETVSIEVKEVVKPLLVSTLGEKSGKGLKTCKSPGRKS
KESSPKGRSSSASSPPKKEHHHHHHHSESPKAPVPLLPPLPPPPPEPESSEDPTSPPEPQDLS
SSVCKEEKMPRGGSLESDGCPKEPAKTQPAVATAATAAEKYKHRGEGER

MeCP2 Triple Mutant (D260A,T299A,S464A) – Yellow=GST tag, Blue= thrombin site, Red=point mutant

MSPILGYWKIKGLVQPTRLLLEYLEEKYEEHLYERDEGDKWRNKKFELGLEFPNLPYYIDGDVK
LTQSMAIIRYIADKHNMLGGCPKERAEISMLEGAVLDIRYGVSRIAYSKDFETLKVDFLSKLPEML
KMFEDRLCHKTYLNGDHVTHPDFMLYDALDVVLYMDPMCLDAFPKLVCFKKRIEAIPQIDKYLK
SSKYIAWPLQGWQATFGGGDHPPKLVPRGSVAGMLGLREEKSEDQDLQGLKDKPLKFKKVKK
DKKEEKEGKHEPVQPSAHHSAEPAEAGKAETSEGSGSAPAVPEASASPKQRRSIIRDRGPMY
DDPTLPEGWTRKLKQRKSGRSAGKYDVYLINPQGKAFRSKVELIAYFEKVGDTSLDPNDFDFT
VTGRGSPSRREQKPPKKPKSPKAPGTGRGRGRPKGSGTTRPKAATSEGVQVKRVLEKSPGK
LLVKMPFQTSPGGKAEGGGATTSTQVMVIKRPGRKRKAEAAPQAIPKKRGRKPGSVVAAAAA
EAKKKAVKESSIRSVQEAVLPIKKRKTRETVSIEVKEVVKPLLVSTLGEKSGKGLKTCKSPGRKS
KESSPKGRSSSASSPPKKEHHHHHHHSESPKAPVPLLPPLPPPPPEPESSEDPTSPPEPQDLS
SSVCKEEKMPRGGSLESDGCPKEPAKTQPAVATAATAAEKYKHRGEGERKDIVSASMPRPNR
EEPVDSRTPVTER

SIRT6:

6XHis-Thrombin-SIRT6 WT: Highlight = 6XHis and thrombin site, in pet28a plasmid

MGSSHHHHHHSSGLVPRGSVNYAAGLSPYADKGKCGLPEIFDPPEELERKVWELARLVWQSS
SVVFHTGAGISTASGIPDFRGPHGVWTMEERGLAPKFDTTFESARPTQTHMALVQLERVGLLR
FLVSQNVDGLHVRSGFPRDKLAELHGNMFVEECAKCKTQYVRDTVVGTMGLKATGRLCTVAK

ARGLRACRGELRDTILDWEDSLPDRDLALADEASRNADLSITLGTSLQIRPSGNLPLATKRRGG
RLVIVNLQPTKHDRHADLRIHGYVDEVMTRLMKHLGLEIPAWDGPRVLERALPPLPRPPTPKLE
PKEESPTRINGSIPAGPKQEPCAQHNGSEPASPKRERPTSPAPHRPPKRVKAKAVPS

6XHis-Thrombin-SIRT6 ΔN24 (25-355): Highlight = 6XHis and thrombin site, in pet28a plasmid

MGSSHHHHHHSSGLVPRGDPPEELERKVWELARLVWQSSSVVFHTGAGISTASGIPDFRGPH
GVWTMEERGLAPKFDTTFESARPTQTHMALVQLERVGLLRFLVSQNVDGLHVRSGFPRDKLA
ELHGNMFVEECAKCKTQYVRDTVVGTMGLKATGRLCTVAKARGLRACRGELRDTILDWEDSL
PDRDLALADEASRNADLSITLGTSLQIRPSGNLPLATKRRGGRLVIVNLQPTKHDRHADLRIHGY
VDEVMTRLMKHLGLEIPAWDGPRVLERALPPLPRPPTPKLEPKEESPTRINGSIPAGPKQEPCA
QHNGSEPASPKRERPTSPAPHRPPKRVKAKAVPS

6XHis-Thrombin-SIRT6 ΔC298 (2-297): Highlight = 6XHis and thrombin site, in pet28a plasmid

MGSSHHHHHHSSGLVPRGSVNYAAGLSPYADKGKCGLPEIFDPPEELERKVWELARLVWQSS
SVVFHTGAGISTASGIPDFRGPHGVWTMEERGLAPKFDTTFESARPTQTHMALVQLERVGLLR
FLVSQNVDGLHVRSGFPRDKLAELHGNMFVEECAKCKTQYVRDTVVGTMGLKATGRLCTVAK
ARGLRACRGELRDTILDWEDSLPDRDLALADEASRNADLSITLGTSLQIRPSGNLPLATKRRGG
RLVIVNLQPTKHDRHADLRIHGYVDEVMTRLMKHLGLEIPAWDGPRVLERALPPLPRPPTPKLE

6XHis-Thrombin-SIRT6 ΔN24ΔC298 (25-297): Highlight = 6XHis and thrombin site, in pet28a plasmid

MGSSHHHHHHSSGLVPRGDPPEELERKVWELARLVWQSSSVVFHTGAGISTASGIPDFRGPH
GVWTMEERGLAPKFDTTFESARPTQTHMALVQLERVGLLRFLVSQNVDGLHVRSGFPRDKLA
ELHGNMFVEECAKCKTQYVRDTVVGTMGLKATGRLCTVAKARGLRACRGELRDTILDWEDSL
PDRDLALADEASRNADLSITLGTSLQIRPSGNLPLATKRRGGRLVIVNLQPTKHDRHADLRIHGY
VDEVMTRLMKHLGLEIPAWDGPRVLERALPPLPRPPTPKLE

SIRT6 WT (2-355):, in pet28a plasmid

SVNYAAGLSPYADKGKCGLPEIFDPPEELERKVWELARLVWQSSSVVFHTGAGISTASGIPDFR
GPHGVWTMEERGLAPKFDTTFESARPTQTHMALVQLERVGLLRFLVSQNVDGLHVRSGFPRD
KLAELHGNMFVEECAKCKTQYVRDTVVGTMGLKATGRLCTVAKARGLRACRGELRDTILDWE
DSLPDRDLALADEASRNADLSITLGTSLQIRPSGNLPLATKRRGGRLVIVNLQPTKHDRHADLRI
HGYVDEVMTRLMKHLGLEIPAWDGPRVLERALPPLPRPPTPKLEPKEESPTRINGSIPAGPKQE
PCAQHNGSEPASPKRERPTSPAPHRPPKRVKAKAVPS

SIRT6 H133Y (2-355): Underline= H133 to Y mutation, in pet28a plasmid

SVNYAAGLSPYADKGKCGLPEIFDPPEELERKVWELARLVWQSSSVVFHTGAGISTASGIPDFR
GPHGVWTMEERGLAPKFDTTFESARPTQTHMALVQLERVGLLRFLVSQNVDGLHVRSGFPRD
KLAELYGNMFVEECAKCKTQYVRDTVVGTMGLKATGRLCTVAKARGLRACRGELRDTILDWE
DSLPDRDLALADEASRNADLSITLGTSLQIRPSGNLPLATKRRGGRLVIVNLQPTKHDRHADLRI
HGYVDEVMTRLMKHLGLEIPAWDGPRVLERALPPLPRPPTPKLEPKEESPTRINGSIPAGPKQE
PCAQHNGSEPASPKRERPTSPAPHRPPKRVKAKAVPS

SIRT6 ΔC298 (2-297): in pet28a plasmid

SVNYAAGLSPYADKGKCGLPEIFDPPEELERKVWELARLVWQSSSVVFHTGAGISTASGIPDFR
GPHGVWTMEERGLAPKFDTTFESARPTQTHMALVQLERVGLLRFLVSQNVDGLHVRSGFPRD
KLAELHGNMFVEECAKCKTQYVRDTVVGTMGLKATGRLCTVAKARGLRACRGELRDTILDWE
DSLPDRDLALADEASRNADLSITLGTSLQIRPSGNLPLATKRRGGRLVIVNLQPTKHDRHADLRI
HGYVDEVMTRLMKHLGLEIPAWDGPRVLERALPPLPRPPTPKLE

NLK WT: (1-527): Highlight = FLAG tag, in bacmid

MSLCGARANAKMMAAYNGGTSAAAAGHHHHHHHHLPHLPPPHLHHHHHPQHHLHPGSAAAV
HPVQQHTSSAAAAAAAAAAAAAAMLNPGQQQPYFPSPAPGQAPGPAAAAPAQVQAAAAATVK
AHHHQHSHHPQQQLDIEPDRPIGYGAFGVVWSVTDPRDGKRVALKKMPNVFQNLVSCKRVFR
ELKMLCFFKHDNVLSALDILQPPHIDYFEEIYVVTELMQSDLHKIIVSPQPLSSDHVKVFLYQILRG
LKYLHSAGILHRDIKPGNLLVNSNCVLKICDFGLARVEELDESRHMTQEVVTQYYRAPEILMGSR
HYSNAIDIWSVGCIFAELLGRRILFQAQSPIQQLDLITDLLGTPSLEAMRTACEGAKAHILRGPHK
QPSLPVLYTLSSQATHEAVHLLCRMLVFDPSKRISAKDALAHPYLDEGRLRYHTCMCKCCFSTS
TGRVYTSDFEPVTNPKFDDTFEKNLSSVRQVKEIIHQFILEQQKGNRVPLCINPQSAAFKSFISS
TVAQPSEMPPSPLVWEGGSGGDYKDDDDK

NLK1 Δ(2-54) H (124/125/126)to A: Underline = HtoA Highlight = FLAG tag in bacmid

MPGSAAAVHPVQQHTSSAAAAAAAAAAAAAAMLNPGQQQPYFPSPAPGQAPGPAAAAPAQVQ
AAAAATVKAAAAQHSHHPQQQLDIEPDRPIGYGAFGVVWSVTDPRDGKRVALKKMPNVFQNL
VSCKRVFRELKMLCFFKHDNVLSALDILQPPHIDYFEEIYVVTELMQSDLHKIIVSPQPLSSDHVK
VFLYQILRGLKYLHSAGILHRDIKPGNLLVNSNCVLKICDFGLARVEELDESRHMTQEVVTQYYR
APEILMGSRHYSNAIDIWSVGCIFAELLGRRILFQAQSPIQQLDLITDLLGTPSLEAMRTACEGAK
AHILRGPHKQPSLPVLYTLSSQATHEAVHLLCRMLVFDPSKRISAKDALAHPYLDEGRLRYHTC
MCKCCFSTSTGRVYTSDFEPVTNPKFDDTFEKNLSSVRQVKEIIHQFILEQQKGNRVPLCINPQ
SAAFKSFISSTVAQPSEMPPSPLVWEGGSGGDYKDDDDK

BAF170 WT (1-1214): Highlight = 3XFLAG tag, in bacmid

MAVRKKDGGPNVKYYEAADTVTQFDNVRLWLGKNYKKYIQAEPPTNKSLSSLVVQLLQFQ
EEVFGKHVSNAPLTKLPIKCFLDFKAGGSLCHILAAAYKFKSDQGWRRYDFQNPSRMDRN
VEMFMTIEKSLVQNNCLSRPNIFLCPEIEPKLLGKLKDIIKRHQGTVTEDKNNASHVVYP
VPGNLEEEEWVRPVMKRDKQVLLHWGYYPDSYDTWIPASEIEASVEDAPTPEKPRKVHAK
WILDTDTFNEWMNEEDYEVNDDKNPVSRRKKISAKTLTDEVNSPDSDRRDKKGGNYKKRK
RSPSPSPTPEAKKKNAKKGPSTPYTKSKRGHREEEQEDLTKDMDEPSPVPNVEEVTLPKT
VNTKKDSESAPVKGGTMTDLDEQEDESMETTGKDEDENSTGNKGEQTKNPDLHEDNVTEQ
THHIIIPSYAAWFDYNSVHAIERRALPEFFNGKNKSKTPEIYLAYRNFMIDTYRLNPQEY
LTSTACRRNLAGDVCAIMRVHAFLEQWGLINYQVDAESRPTPMGPPPTSHFHVLADTPSG
LVPLQPKTPQQTSASQQMLNFPDKGKEKPTDMQNFGLRTDMYTKKNVPSKSKAAASATRE
WTEQETLLLLEALEMYKDDWNKVSEHVGSRTQDECILHFLRLPIEDPYLEDSEASLGPLA
YQPIPFSQSGNPVMSTVAFLASVVDPRVASAAAKSALEEFSKMKEEVPTALVEAHVRKVE
EAAKVTGKADPAFGLESSGIAGTTSDEPERIEESGNDEARVEGQATDEKKEPKEPREGGG
AIEEEAKEKTSEAPKKDEEKGKEGDSEKESEKSDGDPIVDPEKEKEPKEGQEEVLKEVVE
SEGERKTKVERDIGEGNLSTAAAAALAAAAVKAKHLAAVEERKIKSLVALLVETQMKKLE
IKLRHFEELETIMDREREALEYQRQQLLADRQAFHMEQLKYAEMRARQQHFQQMHQQQQQ
PPPALPPGSQPIPPTGAAGPPAVHGLAVAPASVVPAPAGSGAPPGSLGPSEQIGQAGSTA
GPQQQQPAGAPQPGAVPPGVPPPGPHGPSPFPNQQTPPSMMPGAVPGSGHPGVAGNAPLG
LPFGMPPPPPPPAPSIIPFGSLADSISINLPAPPNLHGHHHHLPFAPGTLPPPNLPVSMA

NPLHPNLPATTTMPSSLPLGPGLGSAAAQSPAIVAAVQGNLLPSASPLPDPGTPLPPDPT
APSPGTVTPVPPPQEFGGDYKDDDDKGGSDYKDDDDKGGSDYKDDDDK

BAF170 4XHtoG: Underline = 4XHtoG Highlight = 3XFLAG tag, in bacmid

MAVRKKDGGPNVKYYEAADTVTQFDNVRLWLGKNYKKYIQAEPPTNKSLSSLVVQLLQFQ
EEVFGKHVSNAPLTKLPIKCFLDFKAGGSLCHILAAAYKFKSDQGWRRYDFQNPSRMDRN
VEMFMTIEKSLVQNNCLSRPNIFLCPEIEPKLLGKLKDIIKRHQGTVTEDKNNASHVVYP
VPGNLEEEEWVRPVMKRDKQVLLHWGYYPDSYDTWIPASEIEASVEDAPTPEKPRKVHAK
WILDTDTFNEWMNEEDYEVNDDKNPVSRRKKISAKTLTDEVNSPDSDRRDKKGGNYKKRK
RSPSPSPTPEAKKKNAKKGPSTPYTKSKRGHREEEQEDLTKDMDEPSPVPNVEEVTLPKT
VNTKKDSESAPVKGGTMTDLDEQEDESMETTGKDEDENSTGNKGEQTKNPDLHEDNVTEQ
THHIIIPSYAAWFDYNSVHAIERRALPEFFNGKNKSKTPEIYLAYRNFMIDTYRLNPQEY
LTSTACRRNLAGDVCAIMRVHAFLEQWGLINYQVDAESRPTPMGPPPTSHFHVLADTPSG
LVPLQPKTPQQTSASQQMLNFPDKGKEKPTDMQNFGLRTDMYTKKNVPSKSKAAASATRE
WTEQETLLLLEALEMYKDDWNKVSEHVGSRTQDECILHFLRLPIEDPYLEDSEASLGPLA
YQPIPFSQSGNPVMSTVAFLASVVDPRVASAAAKSALEEFSKMKEEVPTALVEAHVRKVE
EAAKVTGKADPAFGLESSGIAGTTSDEPERIEESGNDEARVEGQATDEKKEPKEPREGGG
AIEEEAKEKTSEAPKKDEEKGKEGDSEKESEKSDGDPIVDPEKEKEPKEGQEEVLKEVVE
SEGERKTKVERDIGEGNLSTAAAAALAAAAVKAKHLAAVEERKIKSLVALLVETQMKKLE
IKLRHFEELETIMDREREALEYQRQQLLADRQAFHMEQLKYAEMRARQQHFQQMHQQQQQ
PPPALPPGSQPIPPTGAAGPPAVHGLAVAPASVVPAPAGSGAPPGSLGPSEQIGQAGSTA
GPQQQQPAGAPQPGAVPPGVPPPGPHGPSPFPNQQTPPSMMPGAVPGSGHPGVAGNAPLG
LPFGMPPPPPPPAPSIIPFGSLADSISINLPAPPNLHGGGGGLPFAPGTLPPPNLPVSMA
NPLHPNLPATTTMPSSLPLGPGLGSAAAQSPAIVAAVQGNLLPSASPLPDPGTPLPPDPT
APSPGTVTPVPPPQEFGGDYKDDDDKGGSDYKDDDDKGGSDYKDDDDK

ARH3 (14-363): in SUMO-pET30 Highlight = HA tag

AGAARSLSRFRGCLAGALLGDCVGSFYEAHDTVDLTSVLRHVQSLEPDP
GTPGSERTEALYYTD DTAMARALVQSLLAKEAFDEVDMAHRFAQEYK
KDPDRGYGAGVVTVFKKLLNPKCRDVFEPARA QFNGKGSYGNGGAM
RVAGISLAYSSVQDVQKFARLSAQLTHASSLGYNGAILQALAVHLALQGES
SSEHFLKQLLGHMEDLEGDAQSVLDARELGMEERPYSSRLKKIGELLDQASV
TREEVVSELGNGIA AFESVPTAIYCFLRCMEPDPEIPSAFNSLQRTL
IYSISLGGDTDTIATMAGAIAGAYYGMDQVPES WQQSCEGYEETDILAQSLHR
VFQKSAQSLHRVFQKSYPYDVPDYARS

pcDNA3.1-YY1: addgene (Plasmid #104395)
pcDNA3.1-HA-YY1-WT- Pink= linker – Yellow = 3xFLAG tag

MYPYDVPDYAGYPYDVPDYAGYPYDVPDYAGGGGGEFMASGDTLYIATDGSEMPAEIVELHEI
EVETIPVETIETTVVGEEEEEDDDDEDGGGGDHGGGGGHGHAGHHHHHHHHHHHPPMIALQ
PLVTDDPTQVHHHQEVILVQTREEVVGGDDSDGLRAEDGFEDQILIPVPAPAGGDDDYIEQTLV
TVAAAGKSGGGGSSSSGGGRVKKGGGKKSGKKSYLSGGAGAAGGGGADPGNKKWEQKQV
QIKTLEGEFSVTMWSSDEKKDIDHETVVEEQIIGENSPPDYSEYMTGKKLPPGGIPGIDLSDPKQ
LAEFARMKPRKIKEDDAPRTIACPHKGCTKMFRDNSAMRKHLHTHGPRVHVCAECGKAFVES
SKLKRHQLVHTGEKPFQCTFEGCGKRFSLDFNLRTHVRIHTGDRPYVCPFDGCNKKFAQSTNL
KSHILTHAKAKNNQ

pcDNA3.1-HA-YY1- polyHis del(70-80) H(98-100)A

MYPYDVPDYAGYPYDVPDYAGYPYDVPDYAGGGGGEFMASGDTLYIATDGSEMPAEIVELHEI
EVETIPVETIETTVVGEEEEEDDDDEDGGGGDHGGGGGHGHAGPPMIALQPLVTDDPTQVAA
AQEVILVQTREEVVGGDDSDGLRAEDGFEDQILIPVPAPAGGDDDYIEQTLVTVAAAGKSGGG
GSSSSGGGRVKKGGGKKSGKKSYLSGGAGAAGGGGADPGNKKWEQKQVQIKTLEGEFSVT
MWSSDEKKDIDHETVVEEQIIGENSPPDYSEYMTGKKLPPGGIPGIDLSDPKQLAEFARMKPRK
IKEDDAPRTIACPHKGCTKMFRDNSAMRKHLHTHGPRVHVCAECGKAFVESSKLKRHQLVHT
GEKPFQCTFEGCGKRFSLDFNLRTHVRIHTGDRPYVCPFDGCNKKFAQSTNLKSHILTHAKAK
NNQ

pLJM1-3XFLAG-SIRT6 WT – Pink= linker – Yellow = 3xFLAG tag

MSVNYAAGLSPYADKGKCGLPEIFDPPEELERKVWELARLVWQSSSVVFHTGAGISTASGIPD
FRGPHGVWTMEERGLAPKFDTTFESARPTQTHMALVQLERVGLLRFLVSQNVDGLHVRSGFP
RDKLAELHGNMFVEECAKCKTQYVRDTVVGTMGLKATGRLCTVAKARGLRACRGELRDTILD
WEDSLPDRDLALADEASRNADLSITLGTSLQIRPSGNLPLATKRRGGRLVIVNLQPTKHDRHAD
LRIHGYVDEVMTRLMKHLGLEIPAWDGPRVLERALPPLPRPPTPKLEPKEESPTRINGSIPAGPK
QEPCAQHNGSEPASPKRERPTSPAPHRPPKRVKAKAVPSEFGGDYKDDDDKGGSDYKDDDD
KGGSDYKDDDDK

pLJM1-3XFLAG-SIRT6 H133Y – Red =HtoY mutation

MSVNYAAGLSPYADKGKCGLPEIFDPPEELERKVWELARLVWQSSSVVFHTGAGISTASGIPD
FRGPHGVWTMEERGLAPKFDTTFESARPTQTHMALVQLERVGLLRFLVSQNVDGLHVRSGFP
RDKLAELYGNMFVEECAKCKTQYVRDTVVGTMGLKATGRLCTVAKARGLRACRGELRDTILD
WEDSLPDRDLALADEASRNADLSITLGTSLQIRPSGNLPLATKRRGGRLVIVNLQPTKHDRHAD
LRIHGYVDEVMTRLMKHLGLEIPAWDGPRVLERALPPLPRPPTPKLEPKEESPTRINGSIPAGPK
QEPCAQHNGSEPASPKRERPTSPAPHRPPKRVKAKAVPSEFGGDYKDDDDKGGSDYKDDDD
KGGSDYKDDDDK

DNA Sequences:

177 bp ds601 DNA:

CTACTGGTACGGCAGACAGGATGTATATATCTGACACGTGCCTGGAGACTAGGGAGTAAT
CCCCTTGGCGGTTAAAACGCGGGGGACAGCGCGTACGTGCGTTTAAGCGGTGCTAGAGC
TGTCTACGACCAATTGAGCGGCCTCGGCACCGGGATTCTCCAGTATTCGAGGCCGTTC

(Note: Used 177 bp ss601 only for **Figure 3B**.)

147 bp ds601 DNA (Note: used 147 bp only in **Figure S3A**):

ACAGGATGTATATATCTGACACGTGCCTGGAGACTAGGGAGTAATCCCCTTGGCGGTTAAA
ACGCGGGGGACAGCGCGTACGTGCGTTTAAGCGGTGCTAGAGCTGTCTACGACCAATTGA
GCGGCCTCGGCACCGGGATTCTCCAG

Note: For the “plasmid” DNA used in **Figure 3C**, a pET28a plasmid with the TEV-SIRT6(WT) gene was used. The full sequence of that plasmid is below:

CCGCACCAACGCGCAGCCCGGACTCGGTAATGGCGCGCATTGCGCCCAGCGCCATCTGA
TCGTTGGCAACCAGCATCGCAGTGGGAACGATGCCCTCATTCAGCATTTGCATGGTTTGTT
GAAAACCGGACATGGCACTCCAGTCGCCTTCCCGTTCCGCTATCGGCTGAATTTGATTGC
GAGTGAGATATTTATGCCAGCCAGCCAGACGCAGACGCGCCGAGACAGAACTTAATGGGC
CCGCTAACAGCGCGATTTGCTGGTGACCCAATGCGACCAGATGCTCCACGCCCAGTCGCG
TACCGTCTTCATGGGAGAAAATAATACTGTTGATGGGTGTCTGGTCAGAGACATCAAGAAA
TAACGCCGGAACATTAGTGCAGGCAGCTTCCACAGCAATGGCATCCTGGTCATCCAGCGG
ATAGTTAATGATCAGCCCACTGACGCGTTGCGCGAGAAGATTGTGCACCGCCGCTTTACA
GGCTTCGACGCCGCTTCGTTCTACCATCGACACCACCACGCTGGCACCCAGTTGATCGGC
GCGAGATTTAATCGCCGCGACAATTTGCGACGGCGCGTGCAGGGCCAGACTGGAGGTGG
CAACGCCAATCAGCAACGACTGTTTGCCCGCCAGTTGTTGTGCCACGCGGTTGGGAATGT
AATTCAGCTCCGCCATCGCCGCTTCCACTTTTTCCCGCGTTTTCGCAGAAACGTGGCTGGC
CTGGTTCACCACGCGGGAAACGGTCTGATAAGAGACACCGGCATACTCTGCGACATCGTA
TAACGTTACTGGTTTCACATTCACCACCCTGAATTGACTCTCTTCCGGGCGCTATCATGCCA
TACCGCGAAAGGTTTTGCGCCATTCGATGGTGTCCGGGATCTCGACGCTCTCCCTTATGC
GACTCCTGCATTAGGAAGCAGCCCAGTAGTAGGTTGAGGCCGTTGAGCACCGCCGCCGC
AAGGAATGGTGCATGCAAGGAGATGGCGCCCAACAGTCCCCCGGCCACGGGGCCTGCCA
CCATACCCACGCCGAAACAAGCGCTCATGAGCCCGAAGTGGCGAGCCCGATCTTCCCCAT
CGGTGATGTCGGCGATATAGGCGCCAGCAACCGCACCTGTGGCGCCGGTGATGCCGGCC
ACGATGCGTCCGGCGTAGAGGATCGAGATCTCGATCCCGCGAAATTAATACGACTCACTAT
AGGGGAATTGTGAGCGGATAACAATTCCCCTCTAGAAATAATTTTGTTTAACTTTAAGAAGG
AGATATACCATGGGCAGCAGCCATCATCATCATCATCACAGCAGCGGCGAGAATCTGTACT
TCCAGAGTGTGAATTACGCGGCGGGGCTGTCGCCGTACGCGGACAAGGGCAAGTGCGGC
CTCCCGGAGATCTTCGACCCCCCGGAGGAGCTGGAGCGGAAGGTGTGGGAACTGGCGAG
GCTGGTCTGGCAGTCTTCCAGTGTGGTGTTCCACACGGGTGCCGGCATCAGCACTGCCTC
TGGCATCCCCGACTTCAGGGGTCCCCACGGAGTCTGGACCATGGAGGAGCGAGGTCTGG
CCCCCAAGTTCGACACCACCTTTGAGAGCGCGCGGCCCACGCAGACCCACATGGCGCTG
GTGCAGCTGGAGCGCGTGGGCCTCCTCCGCTTCCTGGTCAGCCAGAACGTGGACGGGCT
CCATGTGCGCTCAGGCTTCCCCAGGGACAAACTGGCAGAGCTCCACGGGAACATGTTTGT
GGAAGAATGTGCCAAGTGTAAGACGCAGTACGTCCGAGACACAGTCGTGGGCACCATGG
GCCTGAAGGCCACGGGCCGGCTCTGCACCGTGGCTAAGGCAAGGGGGCTGCGAGCCTG
CAGGGGAGAGCTGAGGGACACCATCCTAGACTGGGAGGACTCCCTGCCCGACCGGGACC
TGGCACTCGCCGATGAGGCCAGCAGGAACGCCGACCTGTCCATCACGCTGGGTACATCG
CTGCAGATCCGGCCCAGCGGGAACCTGCCGCTGGCTACCAAGCGCCGGGGAGGCCGCC
TGGTCATCGTCAACCTGCAGCCCACCAAGCACGACCGCCATGCTGACCTCCGCATCCATG
GCTACGTTGACGAGGTCATGACCCGGCTCATGAAGCACCTGGGGCTGGAGATCCCCGCC
TGGGACGGCCCCCGTGTGCTGGAGAGGGCGCTGCCACCCCTGCCCCGCCCGCCCACCC
CCAAGCTGGAGCCCAAGGAGGAATCTCCCACCCGGATCAACGGCTCTATCCCCGCCGGC
CCCAAGCAGGAGCCCTGCGCCCAGCACAACGGCTCAGAGCCCGCCAGCCCCAAACGGGA
GCGGCCCACCAGCCCTGCCCCCCACAGACCCCCCAAAAGGGTGAAGGCCAAGGCGGTCC
CCAGCTGATGACGAAGCTTGCGGCCGCACTCGAGCACCACCACCACCACCACTGAGATCC
GGCTGCTAACAAAGCCCGAAAGGAAGCTGAGTTGGCTGCTGCCACCGCTGAGCAATAACT
AGCATAACCCCTTGGGGCCTCTAAACGGGTCTTGAGGGGTTTTTTGCTGAAAGGAGGAAC
TATATCCGGATTGGCGAATGGGACGCGCCCTGTAGCGGCGCATTAAGCGCGGCGGGTGT
GGTGGTTACGCGCAGCGTGACCGCTACACTTGCCAGCGCCCTAGCGCCCGCTCCTTTCG
CTTTCTTCCCTTCCTTTCTCGCCACGTTCGCCGGCTTTCCCCGTCAAGCTCTAAATCGGGG
GCTCCCTTTAGGGTTCCGATTTAGTGCTTTACGGCACCTCGACCCCAAAAAACTTGATTAG
GGTGATGGTTCACGTAGTGGGCCATCGCCCTGATAGACGGTTTTTCGCCCTTTGACGTTG

GAGTCCACGTTCTTTAATAGTGGACTCTTGTTCCAAACTGGAACAACACTCAACCCTATCTC
GGTCTATTCTTTTGATTTATAAGGGATTTTGCCGATTTCGGCCTATTGGTTAAAAAATGAGC
TGATTTAACAAAAATTTAACGCGAATTTTAACAAAATATTAACGCTTACAATTTAGGTGGCAC
TTTTCGGGGAAATGTGCGCGGAACCCCTATTTGTTTATTTTTCTAAATACATTCAAATATGTA
TCCGCTCATGAATTAATTCTTAGAAAAACTCATCGAGCATCAAATGAAACTGCAATTTATTCA
TATCAGGATTATCAATACCATATTTTTGAAAAAGCCGTTTCTGTAATGAAGGAGAAAACTCA
CCGAGGCAGTTCCATAGGATGGCAAGATCCTGGTATCGGTCTGCGATTCCGACTCGTCCA
ACATCAATACAACCTATTAATTTCCCCTCGTCAAAAATAAGGTTATCAAGTGAGAAATCACC
ATGAGTGACGACTGAATCCGGTGAGAATGGCAAAAGTTTATGCATTTCTTTCCAGACTTGTT
CAACAGGCCAGCCATTACGCTCGTCATCAAAATCACTCGCATCAACCAAACCGTTATTCATT
CGTGATTGCGCCTGAGCGAGACGAAATACGCGATCGCTGTTAAAAGGACAATTACAAACA
GGAATCGAATGCAACCGGCGCAGGAACACTGCCAGCGCATCAACAATATTTTCACCTGAAT
CAGGATATTCTTCTAATACCTGGAATGCTGTTTTCCCGGGGATCGCAGTGGTGAGTAACCA
TGCATCATCAGGAGTACGGATAAAATGCTTGATGGTCGGAAGAGGCATAAATTCCGTCAGC
CAGTTTAGTCTGACCATCTCATCTGTAACATCATTGGCAACGCTACCTTTGCCATGTTTCAG
AAACAACTCTGGCGCATCGGGCTTCCCATACAATCGATAGATTGTCGCACCTGATTGCCCG
ACATTATCGCGAGCCCATTTATACCCATATAAATCAGCATCCATGTTGGAATTTAATCGCGG
CCTAGAGCAAGACGTTTCCCGTTGAATATGGCTCATAACACCCCTTGTATTACTGTTTATGT
AAGCAGACAGTTTTATTGTTCATGACCAAAATCCCTTAACGTGAGTTTTCGTTCCACTGAGC
GTCAGACCCCGTAGAAAAGATCAAAGGATCTTCTTGAGATCCTTTTTTTCTGCGCGTAATCT
GCTGCTTGCAAACAAAAAAACCACCGCTACCAGCGGTGGTTTGTTTGCCGGATCAAGAGCT
ACCAACTCTTTTTCCGAAGGTAACTGGCTTCAGCAGAGCGCAGATACCAAATACTGTCCTT
CTAGTGTAGCCGTAGTTAGGCCACCACTTCAAGAACTCTGTAGCACCGCCTACATACCTCG
CTCTGCTAATCCTGTTACCAGTGGCTGCTGCCAGTGGCGATAAGTCGTGTCTTACCGGGTT
GGACTCAAGACGATAGTTACCGGATAAGGCGCAGCGGTCGGGCTGAACGGGGGGTTCGT
GCACACAGCCCAGCTTGGAGCGAACGACCTACACCGAACTGAGATACCTACAGCGTGAGC
TATGAGAAAGCGCCACGCTTCCCGAAGGGAGAAAGGCGGACAGGTATCCGGTAAGCGGC
AGGGTCGGAACAGGAGAGCGCACGAGGGAGCTTCCAGGGGGAAACGCCTGGTATCTTTA
TAGTCCTGTCGGGTTTCGCCACCTCTGACTTGAGCGTCGATTTTTGTGATGCTCGTCAGGG
GGGCGGAGCCTATGGAAAAACGCCAGCAACGCGGCCTTTTTACGGTTCCTGGCCTTTTGC
TGGCCTTTTGCTCACATGTTCTTTCCTGCGTTATCCCCTGATTCTGTGGATAACCGTATTAC
CGCCTTTGAGTGAGCTGATACCGCTCGCCGCAGCCGAACGACCGAGCGCAGCGAGTCAG
TGAGCGAGGAAGCGGAAGAGCGCCTGATGCGGTATTTTCTCCTTACGCATCTGTGCGGTA
TTTCACACCGCAATGGTGCACTCTCAGTACAATCTGCTCTGATGCCGCATAGTTAAGCCAG
TATACACTCCGCTATCGCTACGTGACTGGGTCATGGCTGCGCCCCGACACCCGCCAACAC
CCGCTGACGCGCCCTGACGGGCTTGTCTGCTCCCGGCATCCGCTTACAGACAAGCTGTGA
CCGTCTCCGGGAGCTGCATGTGTCAGAGGTTTTCACCGTCATCACCGAAACGCGCGAGGC
AGCTGCGGTAAAGCTCATCAGCGTGGTCGTGAAGCGATTCACAGATGTCTGCCTGTTCATC
CGCGTCCAGCTCGTTGAGTTTCTCCAGAAGCGTTAATGTCTGGCTTCTGATAAAGCGGGCC
ATGTTAAGGGCGGTTTTTTCCTGTTTGGTCACTGATGCCTCCGTGTAAGGGGGATTTCTGT
TCATGGGGGTAATGATACCGATGAAACGAGAGAGGATGCTCACGATACGGGTTACTGATG
ATGAACATGCCCGGTTACTGGAACGTTGTGAGGGTAAACAACTGGCGGTATGGATGCGGC
GGGACCAGAGAAAAATCACTCAGGGTCAATGCCAGCGCTTCGTTAATACAGATGTAGGTGT
TCCACAGGGTAGCCAGCAGCATCCTGCGATGCAGATCCGGAACATAATGGTGCAGGGCG
CTGACTTCCGCGTTTCCAGACTTTACGAAACACGGAAACCGAAGACCATTCATGTTGTTGC
TCAGGTCGCAGACGTTTTGCAGCAGCAGTCGCTTCACGTTCGCTCGCGTATCGGTGATTC
ATTCTGCTAACCAGTAAGGCAACCCCGCCAGCCTAGCCGGGTCCTCAACGACAGGAGCAC
GATCATGCGCACCCGTGGGGCCGCCATGCCGGCGATAATGGCCTGCTTCTCGCCGAAAC
GTTTGGTGGCGGGACCAGTGACGAAGGCTTGAGCGAGGGCGTGCAAGATTCCGAATACC
GCAAGCGACAGGCCGATCATCGTCGCGCTCCAGCGAAAGCGGTCCTCGCCGAAAATGAC
CCAGAGCGCTGCCGGCACCTGTCCTACGAGTTGCATGATAAAGAAGACAGTCATAAGTGC

GGCGACGATAGTCATGCCCCGCGCCCACCGGAAGGAGCTGACTGGGTTGAAGGCTCTCA
AGGGCATCGGTCGAGATCCCGGTGCCTAATGAGTGAGCTAACTTACATTAATTGCGTTGCG
CTCACTGCCCGCTTTCCAGTCGGGAAACCTGTCGTGCCAGCTGCATTAATGAATCGGCCA
ACGCGCGGGGAGAGGCGGTTTGCGTATTGGGCGCCAGGGTGGTTTTTCTTTTCACCAGTG
AGACGGGCAACAGCTGATTGCCCTTCACCGCCTGGCCCTGAGAGAGTTGCAGCAAGCGG
TCCACGCTGGTTTGCCCCAGCAGGCGAAAATCCTGTTTGATGGTGGTTAACGGCGGGATA
TAACATGAGCTGTCTTCGGTATCGTCGTATCCCACTACCGAGATAT
