# Supplementary material for: DNA stimulates the deacetylase SIRT6 to mono-ADP-ribosylate proteins with histidine repeats
Source: J Biol Chem. 2025 Apr 23;301(6):108532. doi: 10.1016/j.jbc.2025.108532 (PMC12167490; doi:10.1016/j.jbc.2025.108532)
Supplement: Supplemental data [file mmc2.zip › Data File S5. Uncropped blots and gels v4.0.docx]

Figure 1 A:

Main Text:

Anti-ADPr

Total Protein

Figure 1 A:

Rep 2:

Anti-ADPr

Total Protein

Figure 1 A:

Rep 3:

Anti-ADPr

Total Protein

Figure 1 B:

Main Text:

Anti-ADPr

Total Protein

Figure 1 B:

Rep 2:

Anti-ADPr

Total Protein

Figure 2 A:

Main Text:

Anti-ADPr

Total Protein

Figure 2 A:

Rep 2:

Figure 2 B:

Main Text:

Anti-ADPr

Total Protein

Figure 2 B:

Rep 2:

Anti-ADPr

Total Protein

Figure 2 C:

Main Text:

Anti-ADPr

Total Protein

Figure 2 C:

Rep 2:

Anti-ADPr

Total Protein

Figure 2 D:

Main Text:

Anti-mADPr

Anti-YY1

Anti-FLAG

Figure 2 D:

Rep 2 (second half of the blot, first half of the blot is used for Figure S2 B):

Anti-mADPr

Anti-YY1

Anti-FLAG

Figure S2 A:

Displayed image:

Anti-ADPr

Total Protein

Figure S2 A:

Rep 2:

Anti-ADPr

Total Protein

Figure S2 B:

Displayed image (first half of the blot):

Anti-mADPr

Anti-YY1

Anti-FLAG

Figure 3 A:

Main Text:

Anti-ADPr (second half of blot technical replicate)

Total Protein (second half of blot technical replicate)

Figure 3 A:

Rep 2:

Anti-ADPr (second half of blot technical replicate)

Total Protein (second half of blot technical replicate)

Figure 3 B:

Main Text:

Anti-ADPr (last 3 lanes not described or displayed in text)

Total Protein (last 3 lanes not described or displayed in text)

Figure 3 B:

Rep 2:

Anti-ADPr (last 3 lanes not described or displayed in text)

Total Protein (last 3 lanes not described or displayed in text)

Figure 3 C:

Main Text:

Anti-ADPr

Total Protein

Figure 3 C:

Rep 2:

Anti-ADPr

Total Protein

Figure 3 D:

Main Text:

Anti-ADPr

Anti-H3

Total Protein

Figure 3 D:

Rep 2:

Anti-ADPr

Anti-H3

Total Protein

Figure 3 E:

Main Text:

Anti-ADPr

Total Protein

Figure 3 E:

Rep 2:

Anti-ADPr

Total Protein

Figure S 3A/B (Figure S3 A on left, Figure S3 B on left)

Main Text:

Anti-ADPr

Total Protein

Figure S 3A/B (Figure S3 A on left, Figure S3 B on left)

Rep 2:

Anti-ADPr

Total Protein

Figure S3 C:

Displayed Image:

Anti-H3K9ac

Anti-6XHis

Figure S3 C:

Rep 2:

Anti-H3K9ac

Anti-6XHis

Figure S3 E:

Displayed Image:

EtBr Stain

Figure S3 E:

Rep 2:

EtBr Stain

Figure S3 E:

Rep 3:

EtBr Stain

Figure S3 F:

Displayed Image:

Anti-ADPr

Total Protein

Figure S3 F:

Rep 2:

Anti-ADPr

Total Protein

Figure 4 A:

Main Text:

Anti-ADPr

Total Protein

Figure 4 A:

Rep 2:

Anti-ADPr

Total Protein

Figure 4 B:

Main Text:

Anti-ADPr

Total Protein

Figure 4 B:

Rep 2:

Anti-ADPr

Total Protein

Figure 4 C:

Main text on the left, rep 2 on the right:

Anti-ADPr

Total Protein

Figure 4 D:

Main Text:

Anti-ADPr

Anti-6XHis

Anti-SIRT6

Figure 4 D:

Rep 2:

Anti-ADPr

Anti-6XHis

Anti-SIRT6

Figure S3 A:

Figure S4 A

Displayed Image:

Anti-ADPr

Total Protein

*For both blots, the right half (8 lanes) is the experiment referenced in the manuscript.

Figure S4 A:

Rep 2:

Anti-ADPr

Total Protein

*For both images, the right half (8 lanes) is the experiment referenced in the manuscript.

Figure S4 E

Main Text:

Anti-ADPr

Total Protein

*Band below SIRT6 in reaction 4 and 8 is ARH3

Figure S4 E:

Rep 2:

Anti-ADPr

Total Protein

*Band below SIRT6 in reaction 4 and 8 is ARH3

Figure S4 F

Main Text:

Anti-ADPr

Total Protein

Figure S4 F

Rep 2:

Anti-ADPr

Total Protein

Figure S1 .

Gel for S1 (D,B,C)

Gel for S1 (A,E)

Gel for S1 (F)

Gel for second part of S1 (D)

Figure S6 B.

*The samples to the right are other batches of WT MNs

*The sample to the left is the same H3K9ac MN sample with a higher loading.
